# ATM-deficiency increases genomic instability and metastatic potential in a mouse model of pancreatic cancer

**DOI:** 10.1038/s41598-017-11661-8

**Published:** 2017-09-11

**Authors:** Yiannis Drosos, David Escobar, Ming-Yi Chiang, Kathryn Roys, Virginia Valentine, Marc B. Valentine, Jerold E. Rehg, Vaibhav Sahai, Lesa A. Begley, Jianming Ye, Leena Paul, Peter J. McKinnon, Beatriz Sosa-Pineda

**Affiliations:** 1 Department of Genetics, St. Jude Children’s Research Hospital, Memphis, TN United States; 2 Department of Medicine and the Robert H. Lurie Comprehensive Cancer Center, Northwestern University Feinberg School of Medicine, Chicago, IL United States; 3 Department of Cytogenetics, St. Jude Children’s Research Hospital, Memphis, TN United States; 4 Department of Pathology, St. Jude Children’s Research Hospital, Memphis, TN United States; 5 Department of Internal Medicine, University of Michigan, Ann Arbor, MI United States; 6 Department of General Surgery, University of Michigan, Ann Arbor, MI United States

## Abstract

Germline mutations in *ATM* (encoding the DNA-damage signaling kinase, ataxia-telangiectasia-mutated) increase Familial Pancreatic Cancer (FPC) susceptibility, and *ATM* somatic mutations have been identified in resected human pancreatic tumors. Here we investigated how *Atm* contributes to pancreatic cancer by deleting this gene in a murine model of the disease expressing oncogenic Kras (Kras^G12D^). We show that partial or total ATM deficiency cooperates with Kras^G12D^ to promote highly metastatic pancreatic cancer. We also reveal that ATM is activated in pancreatic precancerous lesions in the context of DNA damage and cell proliferation, and demonstrate that ATM deficiency leads to persistent DNA damage in both precancerous lesions and primary tumors. Using low passage cultures from primary tumors and liver metastases we show that ATM loss accelerates Kras-induced carcinogenesis without conferring a specific phenotype to pancreatic tumors or changing the status of the tumor suppressors p53, p16^Ink4a^ and p19^Arf^. However, ATM deficiency markedly increases the proportion of chromosomal alterations in pancreatic primary tumors and liver metastases. More importantly, ATM deficiency also renders murine pancreatic tumors highly sensitive to radiation. These and other findings in our study conclusively establish that ATM activity poses a major barrier to oncogenic transformation in the pancreas via maintaining genomic stability.

## Introduction

Invasive pancreatic ductal adenocarcinoma (PDAC) is one of the most lethal solid malignancies. These tumors often arise from precancerous lesions called Pancreatic Intraepithelial Neoplasias or PanINs. Mutational activation of Kras is nearly universal in PanINs and PDAC, whereas inactivation of the tumor suppressors *p16* ^*Ink4a*^, *p14* ^*ARF*^ (*p19* ^*Arf*^ in mice), *p53* and *SMAD4* usually accompanies the transition from PanINs to PDAC^1^. Not surprisingly, familial cancer syndromes involving germline mutations in *CDKN2A* and *TP53* enhance pancreatic cancer susceptibility. Likewise, the predisposition to pancreatic cancer increases in families with at least 2 affected first-degree relatives and these forms of the disease are classified as Familial Pancreatic Cancer (FPC)^2^. Similar to other cancers, some of the loci responsible for inherited high-risk PDAC include genes involved with DNA repair or chromosomal stability^3^.

DNA damage can occur by multiple routes, including endogenous stimuli, environmental agents or oncogene-induced replication stress^4^. Double-strand breaks (DSBs) are particularly toxic DNA lesions because they can foster mutations and chromosomal rearrangements that compromise genome stability^4, 5^. The DNA damage response (DDR) pathway senses specific DNA lesions, including those arising during replication stress, and orchestrates cellular responses needed to maintain genome integrity. The serine/threonine kinases ATM, DNA-PK (DNA-dependent protein kinase) and ATR (ataxia telangiectasia and Rad3-related) are members of the PIKK (phosphatidylinositol-3-kinase related kinase) family involved in mobilizing cellular responses downstream of DNA damage^6^. ATM is specifically recruited via the Mre11/Rad50/NBS1 (MRN) complex to regions where DSBs occur, and this event initiates ATM auto-phosphorylation and subsequent ATM-dependent phosphorylation of various substrates (including p53) that activate cell cycle checkpoints to induce cell cycle arrest, apoptosis, or senescence^6, 7^. ATM is central to genome stability because its activity prevents DNA damage from being converted to deleterious lesions, including oncogenic chromosomal rearrangements. Moreover, ATM has also been linked to other biological processes unrelated to DNA repair, such as metabolism and stress-responses^8^.

Biallelic mutations in *ATM* cause a severe, debilitating childhood neurodegenerative and immunodeficiency syndrome known as ataxia telangiectasia (A-T)^7^. A-T patients often become afflicted with cancer and their cells display enhanced chromosomal instability, high sensitivity to agents that cause DSBs, and impaired checkpoint activation or defective apoptosis^7^. In agreement with the observation that *ATM* germline mutations (which are prevalent in close to 1% of the population) increase the susceptibility to various types of cancer^9^, recent studies found that about 5% of patients with hereditary pancreatic cancer carry germline-inactivating mutations in this gene^3, 10, 11^. Furthermore, deep-sequencing methods also identified deleterious mutations in *ATM* in human pancreatic tumors classified as ‘genetically unstable’^12^. These and other results postulate that ATM activity poses a barrier to pancreatic cancer progression via maintaining chromosome stability. To test this hypothesis, we deleted *Atm* in pancreatic progenitors of the PDAC mouse model *LSL*-*Kras* ^*G12D*^ *;Ptf1a* ^+/*cre*^ (also known as *KC*)^13^.

## Results

### Lack of ATM accelerates metastatic murine PDAC formation

We generated *Ptf1a* ^+/*cre*^;*ATM* ^*loxP*/+^ (pancreas *Atm*-heterozygous) and *Ptf1a* ^+/*cre*^;*ATM* ^*loxP*/*loxP*^ (pancreas *Atm*-homozygous) breeders that were indistinguishable from their wildtype littermates (Supplementary Figure 1). These mice were intercrossed with *LSL*-*Kras* ^*G12D*^;*ATM* ^*loxP*/+^ mice to produce *LSL*-*Kras* ^*G12D*^;*Ptf1a* ^+/*cre*^, *LSL*-*Kras* ^*G12D*^;*Ptf1a* ^+/*cre*^;*ATM* ^*loxP*/+^ and *LSL*-*Kras* ^*G12D*^;*Ptf1a* ^+/*cre*^;*ATM* ^*loxP*/*loxP*^ offspring, respectively named *KC*, *KCATMΔ*+ and *KCATMΔΔ*. Mice of the 3 genotypes were used in a survival study that was assembled to investigate the effects of ATM deficiency (partial or total) in Kras^G12D^-driven pancreatic tumor formation.

**Figure 1:**
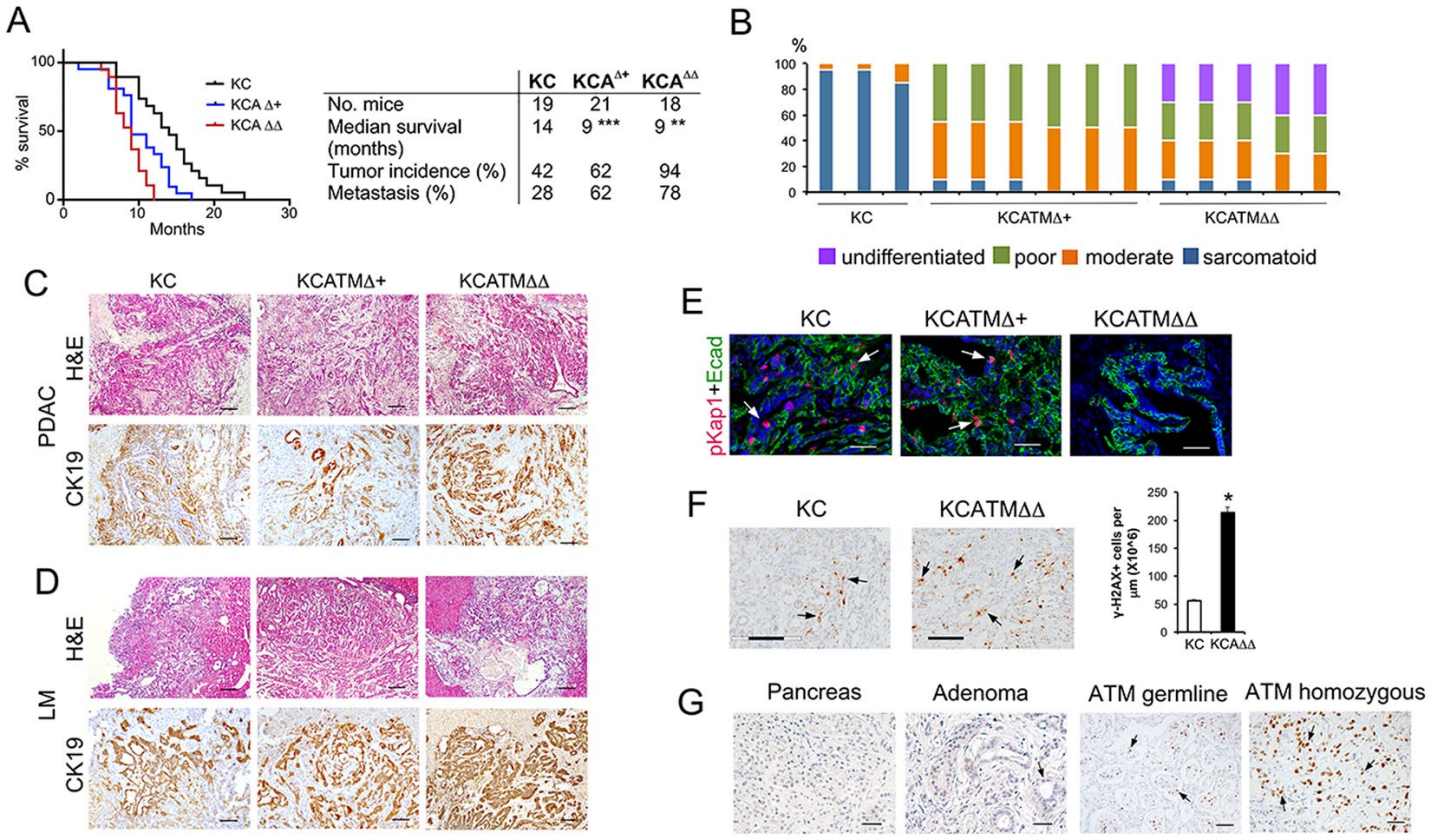
ATM deficiency accelerates metastatic pancreatic cancer formation. (**A**) Kaplan-Meier curves show a dosage-dependent effect of ATM deficiency on survival in mice expressing pancreatic Kras^G12D^ (***P < 0.001 [*KC* vs. *KCATMΔ*+]; **P < 0.01 [*KC* vs. *KCATMΔΔ*] Log rank-test). Partial or total ATM deficiency also increases tumor incidence and the frequency of liver metastasis. (**B**) Summary of tumor histology. **(C)** Representative images of pancreatic tumors stained with H&E or anti-cytokeratin antibodies. **(D)** Representative images of liver metastases stained with H&E or anti-cytokeratin antibodies. **(E)** Cells expressing phospho-Kap1 (red and arrows) are present in some areas of the pancreatic tumor epithelium (stained with anti-Ecadherin antibodies, green) in *KC* and *KCATMΔ*+ mice, and are absent in pancreatic tumors of *KCATMΔΔ* mice. (Scale bars are 40 µm). **(F)** Cells expressing γH2AX foci (arrows) are infrequent in *KC* pancreatic tumors and significantly more abundant in *KCATMΔΔ* pancreatic tumors (*P < 0.05; error bars represent ± SEM values; *n* = 3 individual tumors per genotype, unpaired 2-tailed t-test). **(G)** Human pancreatic specimens stained for γH2AX detection. Prominent γH2AX expression is observed in the tumor specimens carrying *ATM* germline heterozygous mutations and *ATM* somatic homozygous mutations. Scale bars are: 40 µm (**E**), 50 µm (**G**), 100 µm (**C**,**D** [CK-19], **F**) and 200 µm (**C,D**,H&E).

PDAC develops in only a fraction of *KC* mice through a process that is reminiscent to the human disease^13^. Accordingly, roughly 40% of *KC* mice in a C57/NMRI genetic background showed symptoms indicative of tumor formation (Supplementary Table 1) after 1 year of age and succumbed shortly thereafter. Post-mortem analyses revealed that in the *KC* group the incidence of pancreatic tumors and liver metastases was 42% and 28%, respectively (Fig. 1A). This tumor frequency parallels our findings in a previous study using *KC* mice of a similar C57/NMRI mixed background^14^. On the other hand, more than half of the *KCATMΔ*+ and *KCATMΔΔ* mice showed the former symptoms before 1 year of age and displayed an average life expectancy that was almost 5 months shorter than that of *KC* mice (the median survival was: 9 months [*KCATMΔ*+], 9 months [*KCATMΔΔ*], and 14 months [*KC*]; Fig. 1A). Results of post-mortem analysis identified pancreatic tumors in 62% of *KCATMΔ*+ mice and in nearly 100% of *KCATMΔΔ* mice (Fig. 1A). Additionally, this analysis also uncovered liver metastases in 62% of *KCATMΔ*+ mice and in 78% of *KCATMΔΔ* mice (Fig. 1A). These data demonstrate that ATM deficiency synergizes with Kras^G12D^ to promote the formation of highly metastatic pancreatic tumors.

Histopathology analysis and cytokeratin-19 immunostaining of 3 *KC* metastatic tumors and of representative *KC*ATM*Δ*+ (n = 6) and *KCATMΔΔ* (n = 5) tumors identified invasive PDAC with focal or extensive desmoplasia and variable morphology in all specimens (Fig. 1B,C). As has been described, *KC* tumors exhibited mostly sarcomatoid histology whereas *KCATMΔ*+ and *KCATMΔΔ* tumors had lesions with glandular histology and moderate or poor differentiation (Fig. 1B,C). These results indicate that ATM deficiency (partial or total) probably influences the spectrum of PDAC histology in Kras^G12D^-induced pancreatic tumors. Immunostaining results also showed scattered cells expressing the ATM target phospho-Kap1^6, 7^ in both the *KC* and *KCATMΔ*+ pancreatic tumor epithelium (Fig. 1E), and absence of this protein in *KCATMΔΔ* pancreatic tumor epithelial cells (Fig. 1E). This finding indicates that the pancreatic tumors of our *KCATMΔ*+ mice retained a functional copy of *Atm*.

Activation of ATM by DSBs results in phosphorylation of the histone H2A variant H2AX (to generate γH2AX) and localization of this phospho-protein to sites of DNA damage. Since ATM activity has been implicated in oncogene-induced DNA damage responses^4, 15^ we examined the expression of γH2AX in ATM-proficient and ATM-deficient pancreatic tumors of our study group to compare the extent of DNA damage in those tissues. This analysis revealed a few cells expressing γH2AX in tumor specimens from *KC* mice (Fig. 1F), and considerably more cells expressing γH2AX in tumor specimens from *KCATMΔΔ* mice (Fig. 1F). Quantitative results corroborated that the pancreatic tumors of *KCATMΔΔ* mice had significantly more γH2AX^+^ cells than those tumors of *KC* mice (Fig. 1F). Similar analyses performed in human pancreatic specimens showed that γH2AX expression was virtually undetected in both, 1 control (healthy) specimen (Fig. 1G) and 1 specimen harboring a benign adenoma (Fig. 1G). In contrast, cells expressing γH2AX were noticed in focal areas of 1 tumor specimen from a patient with a heterozygous *ATM* germline inactivating mutation (Fig. 1G) and in large areas of 1 human pancreatic tumor that carried homozygous *ATM* somatic inactivating mutations (Fig. 1G). Thus, it is conceivable that ATM signaling is activated in pancreatic tumors of mice and humans to prevent the propagation of unrepaired DNA damage and to preserve genome integrity.

### ATM-proficient and ATM-deficient primary tumors display a ‘pancreatic progenitor/classical’ phenotype and have similar alterations in *Trp53* and *p16*^*INK4A*^/*p19*^*Arf*^ expression

To further understand how ATM loss accelerates the formation of metastatic pancreatic tumors, we established low-passage cell cultures from primary PDACs and liver metastases of all 3 genotypes using published methods^16–18^. PCR analysis of RNA extracted from 3 *KC* cell lines, 3 *KCATMΔ*+ cell lines and 4 *KCATMΔΔ* cell lines showed that all *KC* lines expressed the *Atm* wildtype transcript, all *KCATMΔΔ* lines expressed the deleted (floxed) *Atm* transcript, and all *KCATMΔ*+ cell lines expressed both the wildtype and deleted *Atm* transcript (Supplementary Figure 2). Likewise, FISH analysis revealed that no large deletions involving the *Atm* locus had occurred in *KCATMΔ*+ cell lines generated from PDAC and liver metastases (Supplementary Figure 2). These combined results further indicate that *Atm* loss-of-heterozygosity (LOH) is not a prerequisite for the development of murine metastatic pancreatic tumors that already carry an inactive copy of *Atm*.

**Figure 2:**
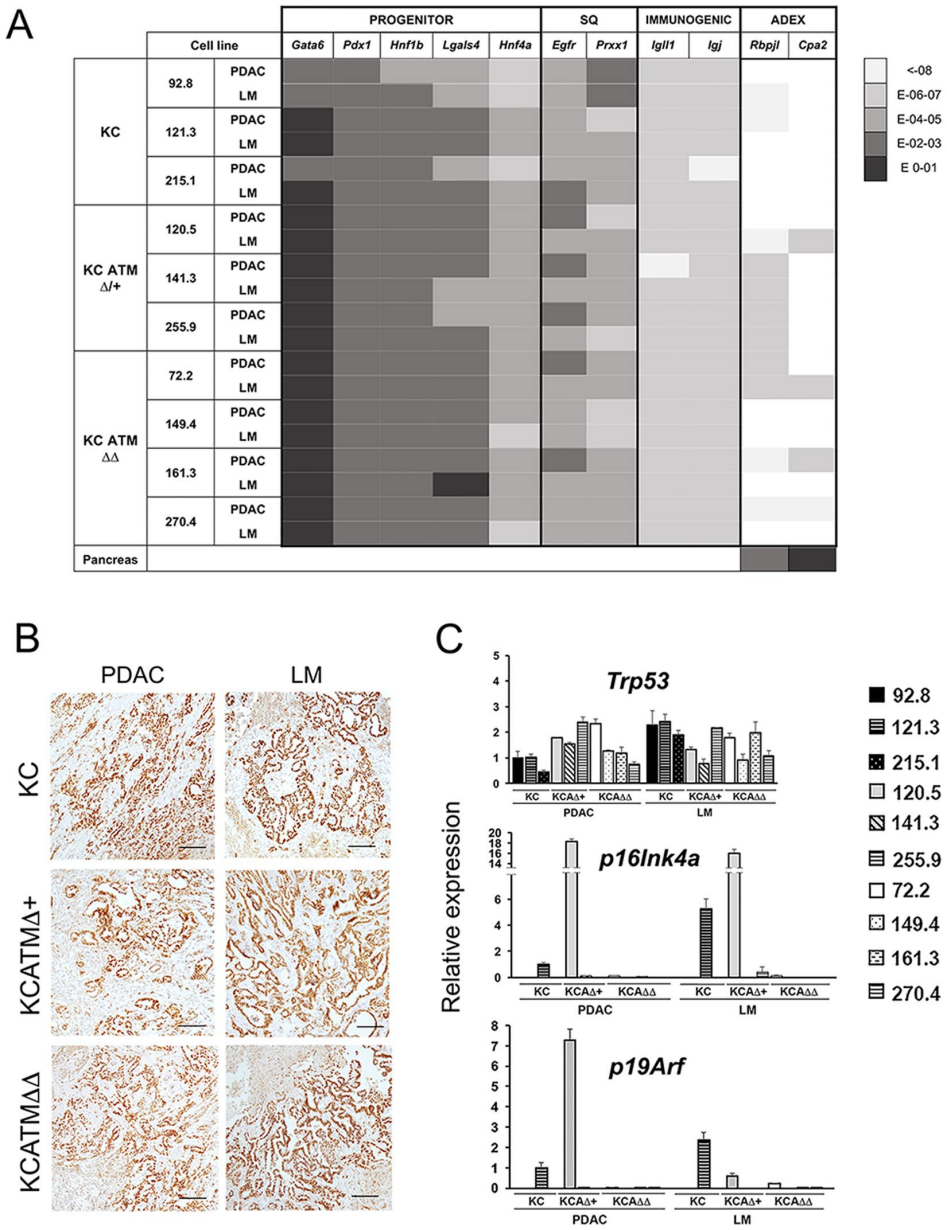
Primary pancreatic tumor cell lines that lack ATM express *Tp53* but not *p16* ^*Ink4a*^/*p19* ^*Arf*^. (**A**) Heat map showing the expression levels of PDA classifiers^19, 20^ in cell lines generated from *KC*, *KCATMΔ*+ and *KCATMΔΔ* primary tumors (PDAC) or liver metastases (LM). The color code (right) indicates the range of expression after normalizing with β-actin. (“Pancreas” is from a P7 mouse pancreas). (**B**) Immunostainig results demonstrate expression of Hnf1β in primary tumors (PDAC) and liver metastases (LM) of all 3 genotypes (Scale bar is 50 µm.) (**C**) QPCR results demonstrate retention of *Tp53* expression in tumor cell lines established from *KC*, *KCATMΔ*+ and *KCATMΔΔ* primary tumors (PDAC) and liver metastases (LM), and loss of *p16* ^*Ink4a*^/*p19* ^*Arf*^ expression in the majority of those cells. (Error bars represent ± SEM values of triplicate experiments).

Two recent studies used genome wide expression methods to classify human pancreatic tumors based on their specific expression profile. In their study, Bailey *et al*.^19^ identified 4 subtypes of PDA that were named squamous, pancreatic progenitor, immunogenic and aberrantly differentiated endocrine exocrine or ADEX. Similarly, Collisson *et al*.^20^ identified 3 tumor subtypes that were named classical, quasimesenchymal and exocrine-like. With the exception of the immunogenic subtype, there is broad overlap amongst the transcriptome classifiers of the pancreatic progenitor/classical, squamous/quasimesenchymal and ADEX/exocrine-like subtypes^19, 20^. We subjected our primary tumor cell lines to qPCR analysis to investigate the expression of transcripts that the former studies used as tumor subtype classifiers. These results showed higher expression of the ‘pancreatic progenitor’ markers *Pdx1*, *Hnf1β* and *Lgals4* ^19^ across all our *KC*, *KCATMΔ*+ and *KCATMΔΔ* tumor cell lines (both from primary tumors and liver metastasis), in comparison to markers of the other subtypes (Fig. 2A and Supplementary Table 2). Moreover, the ‘classical’ tumor marker *Gata6* ^20^ was uniformly expressed at high levels in all *KC*, *KCATMΔ*+ and *KCATMΔΔ* tumor cell lines (Fig. 2A and Supplementary Table 2). Concurrent with the former results, immunostaining analysis of the ‘pancreatic progenitor’ marker Hnf1β showed extensive expression of this transcription factor in both primary tumors and liver metastases of all 3 genotypes (Fig. 2B). Interestingly, our generated tumor cell lines also expressed moderate to low levels of the ‘progenitor’ marker *Hnf4* and the ‘squamous’ markers *Egfr* and *Prxx1* (Fig. 2A). These results agree with the former histopathology results (Fig. 1B,C) showing that all the resected tumors presented variable glandular/sarcomatoid histology. Therefore, we conclude that the Kras^G12D^-driven tumors of our mouse models mainly acquired a pancreatic progenitor/classical phenotype irrespective of the status of ATM.

We also investigated the expression of transcripts encoding the tumor suppressors p53, p16^Ink4a^ and p19^Arf^ ^21^ in our generated pancreatic cancer cell lines using qPCR. This analysis showed similar expression of *Trp53* across all our generated *KC*, *KCATMΔ*+ and *KCATMΔΔ* cell lines (Fig. 2C). In addition, data from exon sequencing using genomic DNA or cDNA showed no mutations in *Trp53* coding regions^22^ in any of the 3 *KCATMΔΔ* PDAC cell lines that we tested (data not shown). On the other hand, qPCR analysis revealed that most tumor cell lines had minimal or no expression of *p16Ink4a*/*p19Arf* (Fig. 2C), with the exception of one *KC* (121.3) cell line and one *KCATMΔ*+ (120.5) cell line. In conclusion, our combined qPCR results indicate that while ATM deficiency accelerates pancreatic cancer formation it does not confer a specific phenotype/genotype to pancreatic tumors.

### Lack of ATM increases chromosomal instability and promotes radiosensitization in pancreatic tumor cells

**Figure 3:**
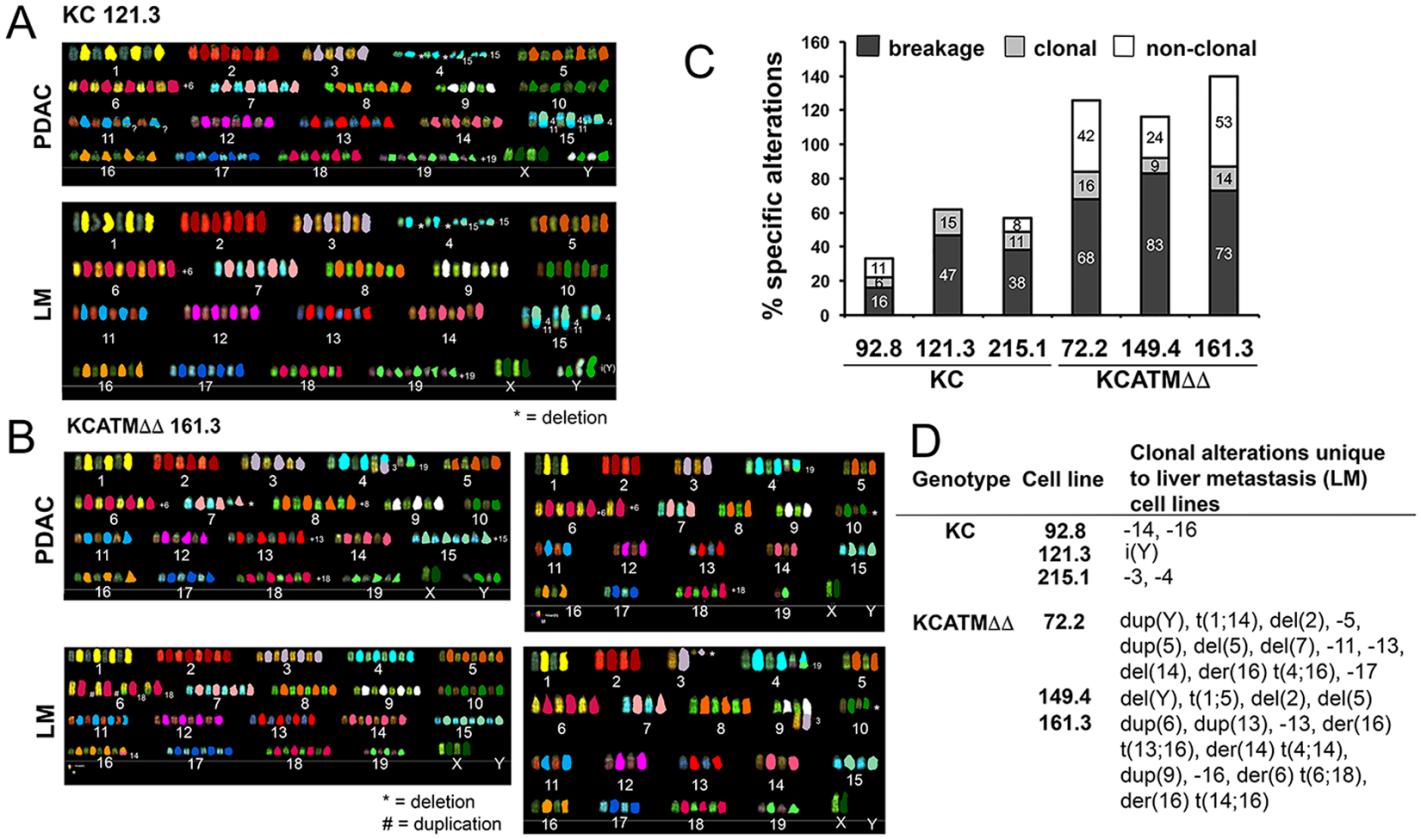
ATM deficiency increases chromosomal alterations in pancreatic cancer cells. (**A**) Representative SKY images showing the chromosomal alterations of low-passage *KC* (121.3) and *KCATMΔΔ* (161.3) cells from both, the primary tumor [PDAC] and a corresponding liver metastasis [LM]). (**B**) Summary of SKY results showing that breakage and non-clonal chromosomal alterations are more frequent in *KCATMΔΔ* cultures than in *KC* cultures. (**C**) SKY data show that clonal alterations unique to liver metastases are more numerous in *KCATMΔΔ* tumor cells than in *KC* tumor cells.

As we determined that ATM-deficient tumors have more widespread DNA damage than ATM-proficient tumors, we examined the extent of chromosomal alterations (an indicator of genome instability) in our established *KC* and *KCATMΔΔ* tumor cell lines using SKY analysis (Fig. 3A,B). SKY results showed similar proportion of clonal alterations (numerical/structural) in 3 individual *KC* primary tumor cell lines and 3 individual *KCATMΔΔ* primary tumor cell lines (6–15% and 9–16% respectively; Fig. 3C). In contrast, the frequency of non-clonal (random) structural alterations was higher in the *KCATMΔΔ* primary tumor cells in comparison to *KC* primary tumor cells (68–83% and 16–47%, respectively; Fig. 3C). In addition, the proportion of breakage type alterations (chromatid gaps/breaks) was also higher in the *KCATMΔΔ* primary tumor cells than in *KCATMΔΔ* primary tumor cells (24–53% and 0–11%, respectively; Fig. 3C). Furthermore, the comparison of clonal alterations between a primary tumor cell line and a liver metastasis cell line from 3 *KC* mice and 3 *KCATMΔΔ* mice underscored more numerical and newly acquired structural abnormalities in the ATM-deficient metastases in comparison to the ATM-proficient metastases (Fig. 3D). These collective SKY results conclusively demonstrate that ATM-deficiency increases chromosomal instability in Kras^G12D^-induced pancreatic tumors.

**Figure 4:**
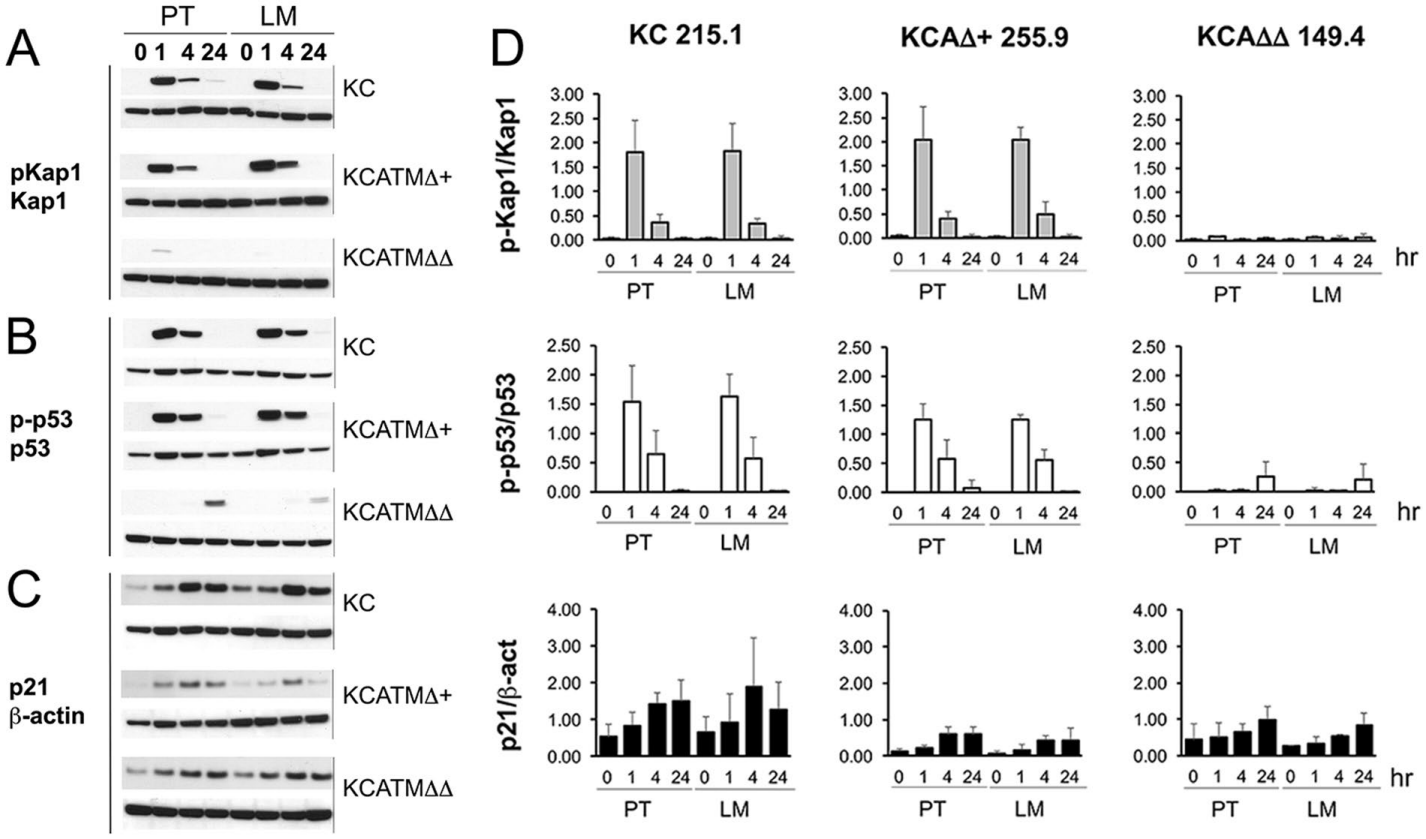
*Atm*-heterozygous pancreatic tumor cells activate ATM signals upon IR exposure. (**A**–**C**) Western blots show induction of phospho-Kap1 (**A**) and phospho-p53 (**B**) proteins in *KC* 215.2 tumor cells and *KCATMΔ*+ 255.9 tumors cells, 1–4 hours post-IR (4Gy). In contrast, phospho-Kap1 is largely undetected (**A**) and phospho-p53 is only slightly induced 24 hours post-IR (**B**) in *KCATMΔΔ* 149.4 tumor cells. (**C**) The expression of p21 increases in tumor cells of all 3 genotypes after exposure to 4Gy. (**D**) Quantitative Western blot results show similar p-Kap1/Kap1 protein ratios in *KC* 215.2 tumor cells and *KCATMΔ*+ 255.9 tumors cells, and lack of p-Kap1 induction in *KCATMΔΔ* 149.4 tumor cells, after 4Gy exposure. This analysis also shows very deficient phospho-p53/p53 ratio in *KCATMΔΔ* 149.4 irradiated cells compared to *KC* 215.2 and *KCATMΔ*+ 255.9 irradiated cells. Quantitative Western blots reveal more efficient induction of p21 in *KC* 215.2 cells than in *KCATMΔ*+ 255.9 and *KCATMΔΔ* 149.4 cells post-irradiation. Bars represent ± SEM values, n = 3 individual hybridizations per genotype). PT: primary tumor. LM: liver metastasis.

Tumors that have defects in DDR components are highly sensitive to agents that cause DNA damage^4^, and published studies showed that *ATM*-nullizygous cells display radiosensitivity^7^. Thus, we investigated if partial or total ATM-deficiency affects the response of pancreatic cells to DNA damage-inducing agents by exposing our *KC* and *KCATMΔΔ* primary tumor cells to X-ray or γ-irradiation (IR). As reported by others^23^, we detected activation of signals downstream of ATM (i.e., induction of the ATM-specific target phospho-Kap1, p53 [Ser-15] phosphorylation, and p21 increase) in mouse embryonic fibroblasts (MEFs) exposed to 4Gy (1–4 hours post-IR) (Supplementary Figure 3). Similarly, those markers were also induced in *KC* 215.1 cells (either from primary PDAC or liver metastasis [LM]) 1–4 hours post-IR (4Gy; Fig. 4A–C). Interestingly, *KCATMΔ*+ 255.9 cells (PDAC and LM-derived) exposed to 4Gy IR also showed phospho-Kap-1 induction and upregulation of phospho-p53 and p21 proteins 1–4 hours post-treatment (Fig. 4A–C), and a similar result was also noticed in 2 additional *KCATMΔ*+ cell lines after irradiation (Supplementary Figure 3). In contrast, *KCATMΔΔ* 149.4 cells (both PDAC and LM) exposed to similar IR doses did not express phospho-Kap1 and only increased phospho-p53 slightly after 24 hours (although p21 induction was similar to that of 215.1 and 255.9 cells; Fig. 4A–C). These results were reproduced using the *KC* cell lines 121.3 and 92.8, and the *KCATMΔΔ* cell lines 72.2 and 161.3 (data not shown) and were validated using quantitative methods (Fig. 4D). Together, these data demonstrate that DNA-damage responses downstream of ATM are functional in *KC* and *KCATMΔ*+ pancreatic tumors, and absent in *KCATMΔΔ* pancreatic tumors.

**Figure 5:**
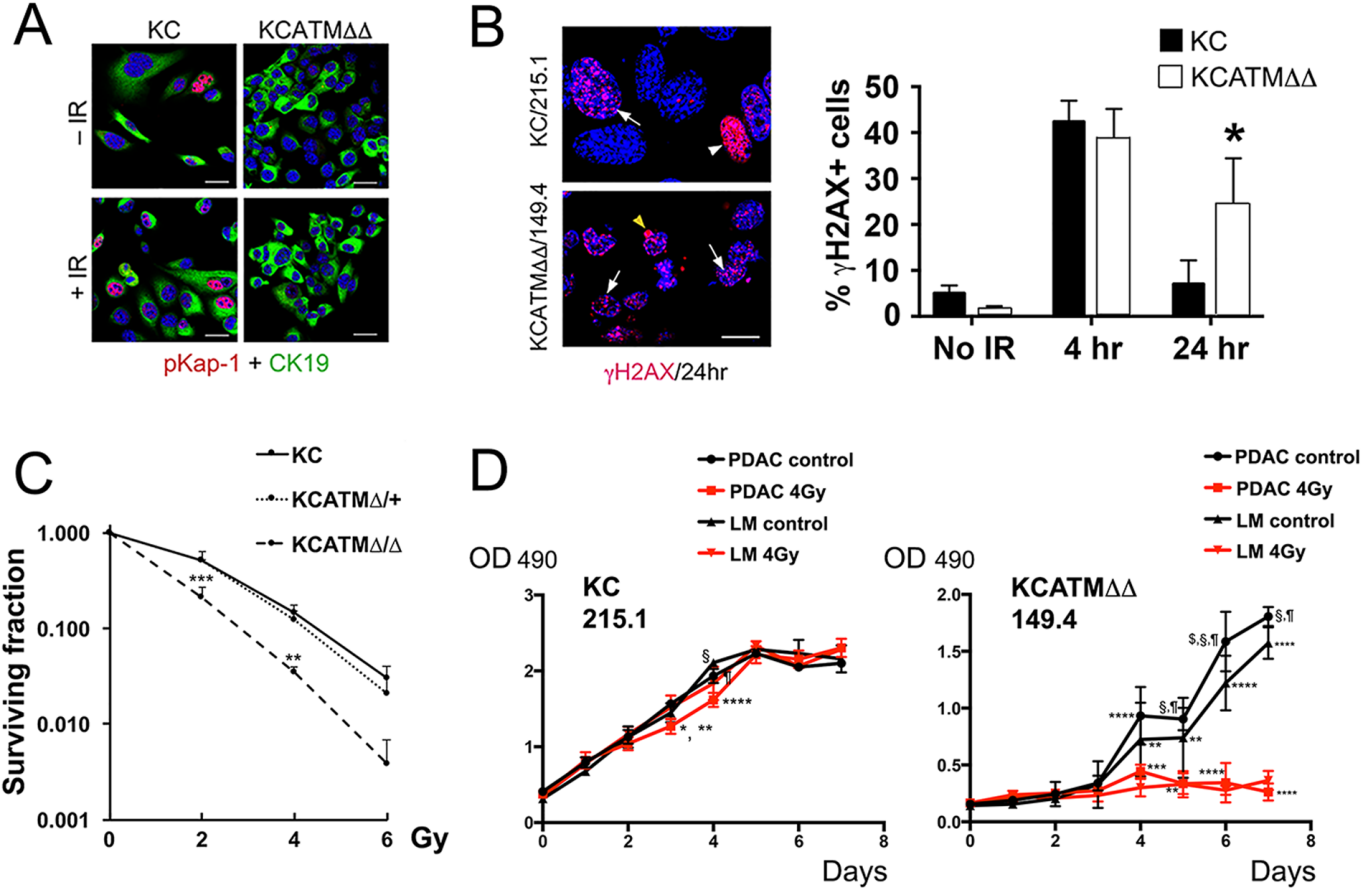
ATM-deficient pancreatic tumor cells are radiosensitive. (**A**) Immunofluorescence analysis shows induction of phospho-Kap1 expression in *KC* 215.2 cells 4 hours after 4Gy exposure and lack of this protein in *KCATMΔΔ* 149.4 cells pre- and post-IR. (**B**) γH2AX foci increase at 4h and diminish 24 h post-IR treatment (4Gy) in *KC* 215.2 cultures. In contrast, γH2AX foci persist in *KCATMΔΔ* 149.4 cultures 24 h post-IR. (A representative γH2AX foci staining is indicated with arrows. The cell showing strong γH2AX staining [arrowheads] is probably apoptotic. *P < 0.05. Error bars represent ± SEM values; n = 3 cell lines, 2-Way ANOVA with Bonferroni’s multiple comparison test). (**C**) Results of clonogenic assay show comparable viability responses of *KC* 215.2 and *KCATMΔ*+ 255.9 tumor cells subjected to different doses of irradiation, and significantly reduced cell viability of *KCATMΔΔ* 149.4 tumor cells after similar IR treatment. (*P* values are from *KC* 215.2 vs. *KCATMΔΔ* 149.4 cell comparisons; **P < 0.01, ***P < 0.001. Error bars represent ± SEM values of triplicate experiments, 2-Way ANOVA with Bonferroni’s multiple comparison test). (**D**) Results of MTT assay show slightly reduced cellular response of *KC* 215.2 tumor cells exposed to 4Gy irradiation (*KC* 215.1: *P < 0.05 [PDAC 4Gy vs. LM 4Gy]; **P < 0.01 [PDAC control vs. 4Gy]; ****P < 0.0001 [PDAC Gy vs. LM ctrl.]; ^¶^ *P* < 0.01 [PDAC control vs. 4Gy]; ^§^ *P* < 0.01 [LM control vs. 4Gy]). MTT results also demonstrate increased sensitization of *KCATMΔΔ* tumor cells upon 4Gy exposure (day 4, **P < 0.01 [LM control vs. 4Gy]; ***P < 0.001 [PDAC control vs. 4Gy], ****P < 0.0001 [PDAC control vs. LM 4Gy]; day 5, **P < 0.01 [PDAC 4Gy vs. LM control and LM control vs. 4Gy]; ^§,¶^ *P* < 0.0001 [PDAC control vs. 4Gy and PDAC control vs. LM 4 Gy]; day 6, ****P < 0.0001 [PDAC 4 Gy. vs. LM control and LM control vs. 4Gy]; ^$^ *P* < 0.05 [PDAC control vs. LM control], ^§^ *P* < 0.0001 [PDAC control vs. LM 4Gy], ^¶^ *P* < 0.0001 [PDAC control vs. 4Gy]; day 7, ****P < 0.0001 [PDAC 4Gy. vs. LM control and LM control vs. 4Gy]; ^§^ *P* < 0.0001 [PDAC control vs. 4Gy], ^¶^ *P* < 0.0001 [PDAC control vs. LM 4Gy]. PDAC: primary tumor cells. LM: liver metastasis cells. *P* values are calculated before the cell cultures are confluent. Error bars represent ± SEM values; n = 3 replicates, 2-Way ANOVA with Bonferroni’s multiple comparison test). Scale bars: 25 µm (**A**), 12.5 µm (**B**).

In agreement with the previous Western blot results, immunostaining analysis revealed expression of phospho-Kap1 in *KC* 215.1 cells exposed to 4Gy IR and lack of this protein in *KCATMΔΔ* 149.4 cells subjected to irradiation (Fig. 5A). In addition, immunostaining with anti- γH2AX antibodies uncovered numerous cells containing γH2AX+ foci in *KCATMΔΔ* 149.4 cultures (Fig. 5B) and less cells showing γH2AX+ foci in *KC* 215.1 cultures (Fig. 5B), 24h post-IR. Quantification of these results corroborated that significantly more γH2AX+ foci persisted in *KCATMΔΔ* 149.4 cells 24h after 4Gy IR exposure in comparison to *KC* 215.1 cultures. This data argued that the ability to repair IR-induced DNA damage is seriously compromised in *ATM* deficient PDAC cells. To further explore this notion, we compared the effects of IR-exposure in the survival of *KC* (215.1), *KCATMΔ*+ (255.9) and *KCATMΔΔ* (149.4) PDAC cells using a clonogenic assay. Our data showed similar survival response to increasing amounts of IR (0–6 Gy, X-ray) between *KC* cells and *KCATMΔ*+ cells (Fig. 5C), and significantly reduced survival of *KCATMΔΔ* cells subjected to the same IR regimen (Fig. 5C) in comparison to *KC* cells. To expand these observations, we used the MTT cell proliferation assay to compare the effects of irradiation between *KC* 215.1 cells (PDAC and LM) and *KCATMΔΔ* 149.4 cells (PDAC and LM). Similar to our previous clonogenic assay results (Fig. 5C), *KC* cells exposed to 4Gy γ-IR displayed radioresistance (Fig. 5D) and *KCATMΔΔ* 149.4 cells showed radiosensitivity (Fig. 5D) in a MTT assay. On the other hand, our MTT results revealed that *KCATMΔ*+ 255.9 PDAC cells are relatively radioresistant (Supplementary Figure 3). In conclusion, these novel results demonstrate that ATM deficiency confers a vulnerability (radiosensitivity) to pancreatic cancer cells that can be exploited therapeutically.

### ATM deficiency increases DNA damage in Kras^G12D^-induced pancreatic precancerous lesions

**Figure 6:**
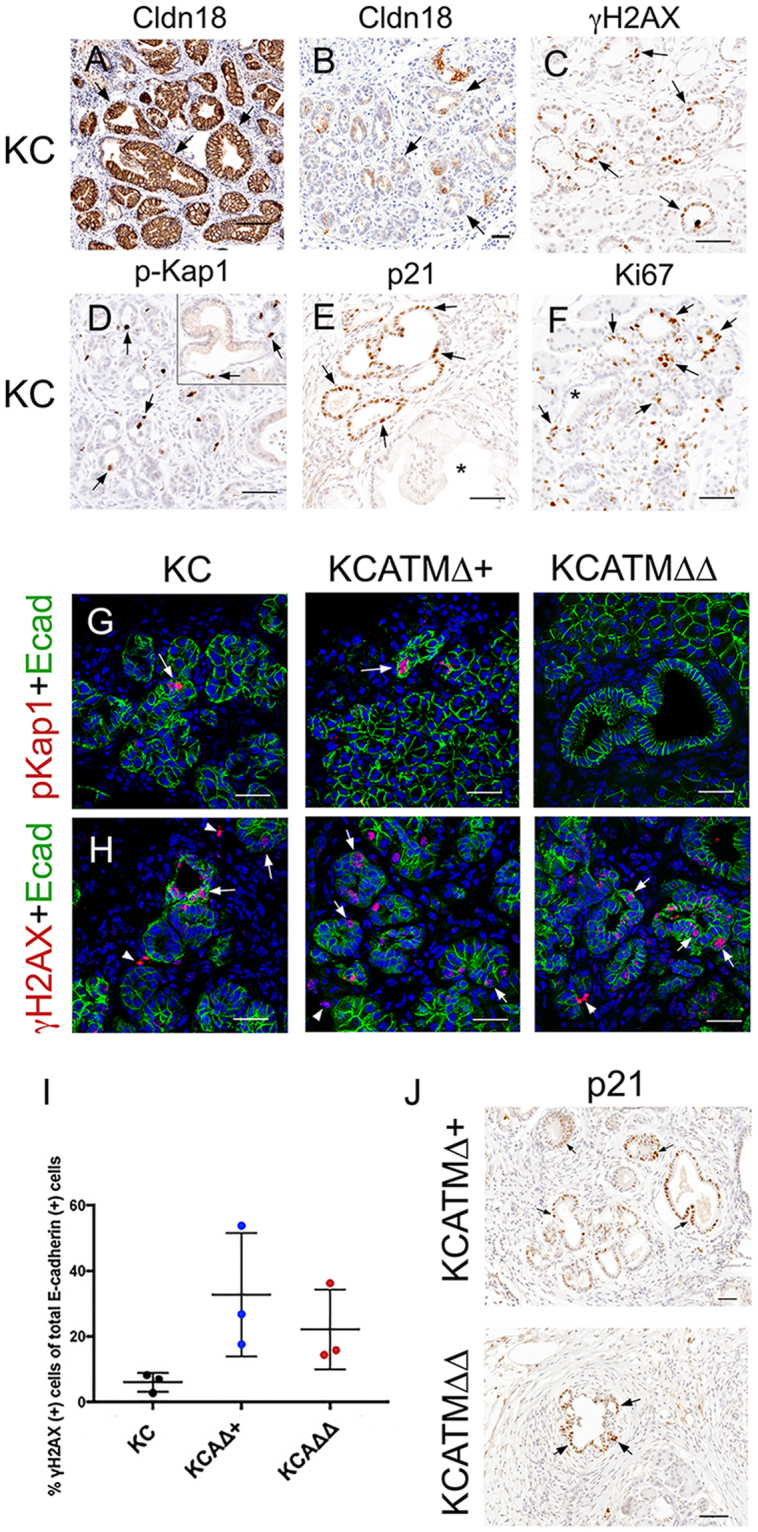
ATM-deficiency increases DNA damage downstream of Kras^G12D^ in pancreatic precancerous lesions. (**A,B**) The abundance of cytoplasm and claudin-18 expression distinguish mPanINs (**A**, arrows) from small ductal lesions (**B**, arrows) in the pancreas of *KC* mice. (**C**–**F**) Markers of DNA damage (γH2AX, **C**), ATM signaling (phospho-Kap1, **D**), p53 activity (p21, **E**), and cell proliferation (Ki67, **F**) are expressed in small ductal lesions (arrows), and to a lesser extent in mPanINs (inset in **D** and asterisks in **E**,**F**), in *KC* pancreata. (**G**) The ATM-target phospho-Kap1 (red, arrows) is expressed in the pancreatic epithelium (Ecadherin^+^, green) of *KC* and *KCATMΔ*+ mice but not in this tissue of *KCATMΔΔ* mice. (**H**) Few cells express the DNA damage indicator γH2AX (red, arrows) in the pancreatic epithelium of *KC* mice. In contrast, γH2AX^+^ cells are more numerous in the pancreatic epithelium of *KCATMΔ*+ and *KCATMΔΔ* mice (arrowheads indicate non-epithelial cells). **(I)**: Quantification of the fraction of Ecadherin^+^ cells that is also positive for γH2AX in *KC*, *KCATMΔ*+ and *KCATMΔΔ* pancreata (*P < 0.05; error bars represent ± SEM values; n = 3–4 individual specimens per genotype, ordinary one-way ANOVA). **(J)** Partial or total ATM deficiency does not affect the expression of p21 (arrows) in pancreatic precancerous lesions. Pancreatic tissues are from 2–4 months old mice. Scale bars: 25 µm (**A**,**B**); 50 µm (**C**–**H**,**J**).

Kras^G12D^-driven murine pancreatic tumors arise from precancerous lesions that include small ductal lesions and mPanINs (Fig. 6A,B), and these epithelial entities are increasingly abundant in the pancreas of *KC* mice after 1 month of age^14, 24–26^. Interestingly, a published study^27^ reported activation of the ATM-CHK2 checkpoint and mounting DNA damage (in the form of γH2AX expression) in early human PanINs. Therefore, we investigated the expression of ATM-downstream effectors in precancerous lesions of *KC* mice using immunostaining methods. We detected expression of γH2AX (Fig. 6C), the specific ATM target phospho-Kap1^6, 7^ (Fig. 6D), and the p53 target p21 (Fig. 6E), in small ductal lesions – and to a lesser extent in mPanINs – in pancreatic tissues of 2-months old *KC* mice. Additionally, we uncovered extensive expression of the proliferation marker Ki67 in small ductal lesions (Fig. 6F), and more limited expression of this marker in mPanINs (Fig. 6F), in 2-months old *KC* pancreata. These collective results demonstrate that ATM signaling is activated in murine pancreatic precancerous lesions in the context of cell proliferation and DNA damage.

The previous results prompted investigating whether, similar to our findings in tumors (Fig. 1F,G), ATM deficiency increases DNA damage in pancreatic precancerous lesions. Immunofluorescence results showed expression of phospho-Kap1 in the pancreatic epithelium (Ecadherin^+^) of 2–4 months old *KC* and *KCATMΔ*+ mice, and lack of this protein in the pancreas of *KCATMΔΔ* littermates (Fig. 6G). This data validated that *Atm* was effectively deleted in *KCATMΔΔ* pancreatic tissues. More importantly, double-immunofluorescence results revealed that ductal epithelial lesions exhibiting γH2AX^+^ foci were more abundant in *KCATMΔ*+ and *KCATMΔΔ* pancreata than in *KC* pancreata (Fig. 6H). Quantification of these results corroborated that γH2AX^+^ cells were more numerous in pancreatic epithelial cells of 2-months old *KCATMΔ*+ and *KCATMΔΔ* mice in comparison to those cells of *KC* littermates (Fig. 6I). These results conclusively establish that ATM activity is necessary to prevent the accumulation of excessive DNA damage in pancreatic precancerous lesions induced by Kras^G12D^. On the other hand, our finding that p21 was expressed in the pancreas of *KCATMΔ*+ and *KCATMΔΔ* mice (Fig. 6J) indicated that ATM activity is dispensable or redundant for the induction of p53-dependent checkpoints in pancreatic precancerous lesions.

### ATM deficiency does not prevent Kras^G12D^-induced senescence but enhances mPanIN formation

Oncogene-induced senescence is a major tumor barrier in human precancerous lesions and it has been suggested that this process follows activation of a robust DDR^28, 29^. On the other hand, the role of ATM in oncogene-induced senescence is controversial. For instance, a published study demonstrated that ATM inhibition prevents Kras-induced senescence in human fibroblasts^30^. In contrast, a separate report showed that lack of ATM does not affect senescence induction in Kras^V12^-driven mouse lung adenomas^31^. These discrepancies prompted us to investigate the effects of ATM deficiency in senescence induction in murine Kras^G12D^-induced pancreatic precancerous lesions.

**Figure 7:**
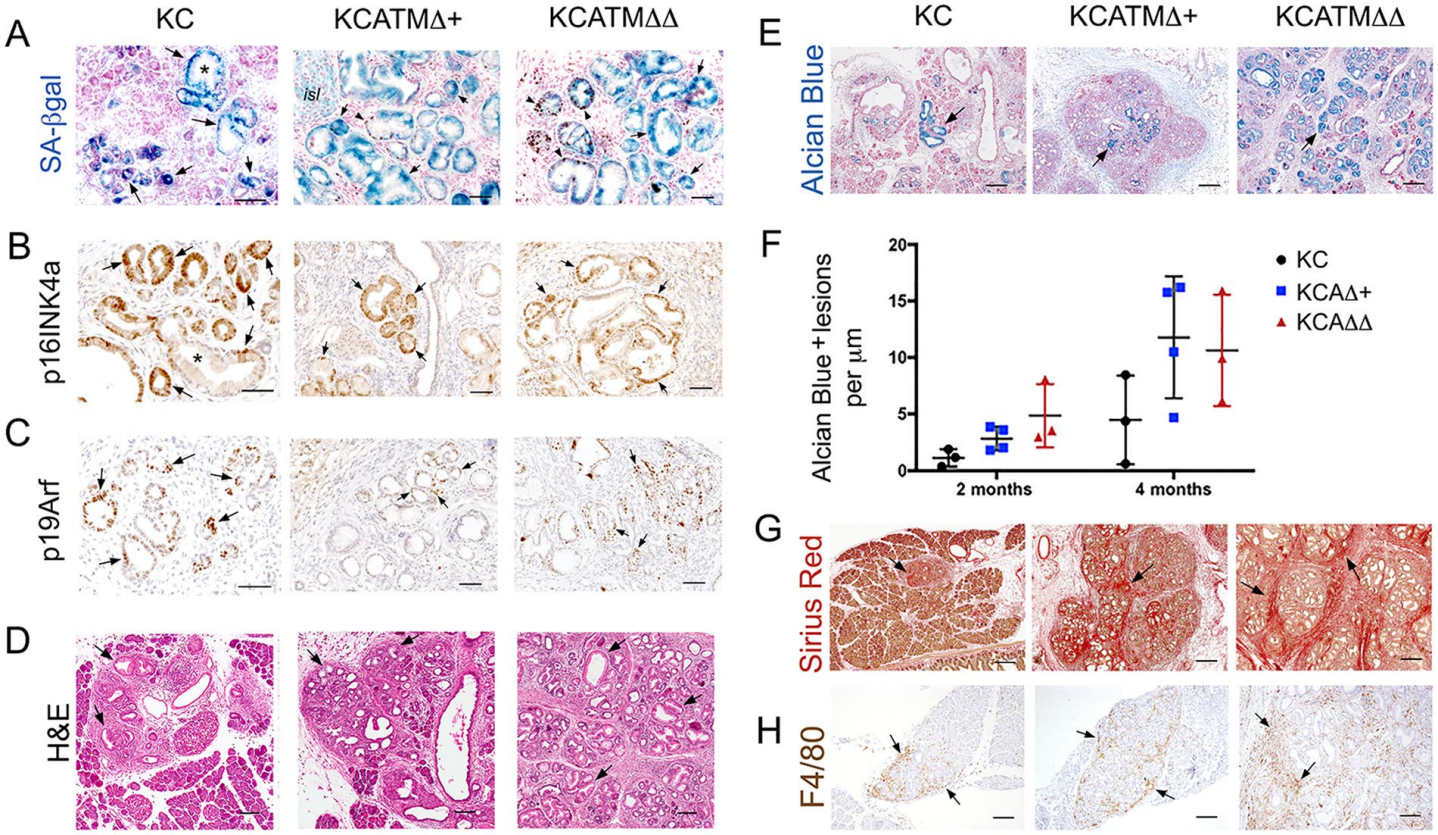
ATM deficiency enhances mPanIN formation but does not prevent Kras^G12D^-induced senescence. (**A**–**C**) Partial or total ATM deficiency does not abolish the induction of senescence (**A**, SA-βgal staining) or the expression of p16^Ink4a^ (**B**) or p19^Arf^ (**C**), in Kras^G12D^-induced pancreatic precancerous lesions. (Arrows indicate small ductal lesions and asterisks indicate mPanINs). (**D**,**E**) ATM deficiency increases areas containing low-grade mPanINs (H&E staining, arrows in (**D**) and Alcian Blue staining, arrows in **E**) in a dosage-dependent manner in Kras^G12D^ pancreata. (**F**) Quantification of Alcian Blue^+^ lesions in 2–4 months old *KC*, *KCATMΔ*+ and *KCATMΔΔ* pancreata demonstrates that low grade mPanINs are more abundant in tissues carrying partial or total ATM-deficiency (*P < 0.05; error bars represent ± SEM values; n = 3–4 individual specimens per genotype, ordinary one-way ANOVA). (**G**,**H**) ATM deficiency increases areas of fibrosis (Sirius red staining of collagen fibrils, arrows in **G**) and areas containing macrophage infiltrates (F4/80 immunostaining, arrows in **H**) in Kras^G12D^ pancreata. Pancreatic specimens are from 2–4 months old mice. Scale bars: 200 µm (**E**,**G**); 100 µm (**D**,**H**); 50 µm (**A**–**C**).

We stained the pancreas of 2–4 months old *KC* mice for SA-βgal detection to identify senescent cells. To complement this analysis, we also stained those tissues with antibodies recognizing p16^Ink4a^ because a previous study reported concomitant expression of p16^Ink4a^ and SA-βgal in mPanINs induced by Kras^V12^ ^32^. We observed broad expression of SA-βgal in pancreatic precancerous epithelial lesions (Fig. 7A) of *KC* mice. In addition, we detected p16^Ink4a^ and p19^Arf^ (the 2 gene products of *Cdkn2a*) protein expression in both small ductal lesions and mPanINs (Fig. 7B,C) in the pancreas of *KC* mice. Interestingly, our SA-βgal staining results showed no differences in the expression of this marker amongst precancerous lesions of *KC*, *KCATMΔ*+ and *KCATMΔΔ* mice (Fig. 7A). Also, both p16^Ink4a^ (Fig. 7B) and p19^Arf^ (Fig. 7C) had comparable expression in pancreatic precancerous lesions of the 3 genotypes. These results demonstrate that ATM activity is dispensable for oncogene-induced senescence in Kras^G12D^-driven pancreatic precancerous lesions. In addition, our finding that precancerous lesions but not pancreatic tumors express p16^Ink4a^ and p19^Arf^ in our mouse models (Fig. 2C) further argues that the checkpoint controlled by these proteins is lost late in pancreas carcinogenesis”.

We also stained the pancreata of our mouse models with H&E and uncovered focal areas harboring ductal epithelial structures surrounded by stroma in those tissues of 2 months old *KC* mice (Fig. 7D), and more extensive areas containing stroma and ductal epithelial lesions in the pancreas of *KCATMΔ*+ and *KCATMΔΔ* littermates (Fig. 7D). To further this observation, the pancreata of 2–4 months old *KC*, *KCATMΔ*+ and *KCATMΔΔ* mice was stained with Alcian Blue^33^ and the mPanIN lesions were counted. Results of this quantitative analysis corroborated that mPanINs were more numerous in the pancreas of both *KCATMΔ*+ and *KCATMΔΔ* mice than in this organ of *KC* littermates (Fig. 7E,F). Likewise, histologic and immunostaining results confirmed that the fibrotic stroma (Fig. 7G) and the macrophage infiltrates (Fig. 7H) were more prominent in the pancreas of *KCATMΔ*+ and *KCATMΔΔ* mice than in this organ of *KC* littermates. These results underscore that partial or total ATM deficiency directly or indirectly enhances Kras^G12D^-driven mPanIN formation and the associated inflammation.

## Discussion

The activation of DNA damage responses downstream of oncogene-induced replication poses a major barrier to cancer progression and contributes to maintain genomic stability^5, 34^. ATM plays an important role in cancer protection because its activity engages the molecular machinery that repairs DSBs^6, 7^. In addition, ATM induces p53-dependent responses that promote cell cycle arrest, senescence or apoptosis to prevent the propagation of excessive DNA damage^5, 15, 35^. In this study we demonstrate that ATM activity is necessary for genome surveillance in the pancreas. First, we show that partial or total ATM deficiency cooperates with oncogenic Kras to promote highly metastatic murine pancreatic cancer. Second, we establish that lack of ATM causes persistent DNA damage in pancreatic tumors of mice and possibly also in pancreatic tumors of humans. Third, we show that ATM activity is necessary in established pancreatic tumors to prevent the accumulation of deleterious DNA lesions and to preserve genome integrity. These and other results suggest a model whereby oncogene-induced replication stress promotes DSBs in pancreatic precancerous cells and elicits activation of an ATM-mediated signaling cascade to repair the damaged DNA. In the absence of ATM activity, DNA damage (in the form of unrepaired DSBs) escalates, new oncogenic mutations are acquired, genomic instability accumulates, and pancreatic cancerous cells emerge. Thus, our study conclusively establishes that ATM’s main tumor suppressor role in the pancreas is to maintain genomic stability. Moreover, the marked differences in the number of clonal alterations uncovered between our *KC* and *KCATMΔΔ* metastatic cell lines indicate that ATM activity restrains PDAC in a locally advanced state, and that once its function is lost the accumulation of genomic instability enhances distant metastasis. This notion concurs with published data showing that human sporadic tumors that exhibit unstable genomes, chemoresistance, and poor prognosis (including those arising in the pancreas), frequently carry *ATM* somatic mutations^9, 32^.

Similar to our study, Russell *et al*.^36^ showed that loss of ATM accelerates Kras^G12D^-induced pancreatic cancer in mice. However, those authors did not report chromosomal alterations or excessive DNA damage in pancreatic tumors that lack ATM. Instead, they concluded that ATM deficiency enhances pancreatic tumor potential by increasing EMT and BMP4 signaling in precancerous lesions^36^. While these parameters were not evaluated in our analysis, a direct role of ATM in EMT or BMP signaling is yet to be proven. Moreover, our SKY results strongly indicate that the primary driver of metastasis in ATM-deficient pancreatic tumors is chromosomal instability. This conclusion concurs with the recognized role of ATM in sensing DNA damage^6, 7^, and with published results showing that aggressive, unstable forms of PDAC often carry somatic *ATM* mutations^32^. Therefore, our hypothesis that ATM activity is key to preserve genomic stability in pancreatic cells under oncogenic stress is well supported. On the other hand, we find intriguing that our generated ATM-deficient and ATM-proficient tumor cell lines shared a similar ‘pancreatic progenitor/classical’ subtype expression profile (including high *Gata6* expression), because those features were found to correlate with better outcome in PDA patients^30, 31^. Thus, it is possible that the increased genomic instability of *ATM* deficient tumors contributes to disable some of the transcriptional networks and gene programs operating in ‘pancreatic progenitor/classical’ tumors. Addressing this and other outstanding questions using genome-wide approaches is necessary to understand how ATM deficiency increases pancreatic metastatic disease.

Our new finding that murine pancreatic precancerous lesions express phospho-Kap1 coincides with similar observations in humans^27^ and argues that ATM is activated very early during Kras^G12D^-driven oncogenesis, probably in response to DSBs induced by replication stress^4, 35^. However, ATM could accomplish functions that do not involve signaling DNA damage in pancreatic precancerous lesions. For instance, ATM activity has been implicated in cellular redox homeostasis independent from its role in DNA damage responses^8^. Also, the activation of DNA damage (by ROS) and ATM signaling was shown to suppress the production of cytokines^37^. A potential regulatory role of ATM in both cellular redox and cytokine production in pancreatic tissues would agree with our finding that both *KCATMΔ*+ and *KCATMΔΔ* mice present increased DNA damage, extensive fibrosis and abundant macrophage infiltrates in the pancreas. Moreover, overabundance of cytokines could enhance a stromal reaction that favors the formation of mPanINs^38, 39^. Investigating these issues should help disclosing how ATM activity protects precancerous pancreatic cells from oncogenic transformation.

The discovery that our generated ATM-deficient pancreatic tumor cells expressed p53 but not p16^Ink4A^/p19^Arf^ concurs with the observation that Kras^G12D^-induced murine pancreatic tumors have frequent loss of p16^Ink4A^ and p19^Arf^ or loss of p53, but not simultaneous loss of the 3 tumor suppressors^21^. Moreover, our findings also agree with a whole-genome sequencing study^22^ showing more frequent mutations or losses of *CDKN2A* (6/9 tumors) than *TP53* (3/9 tumors) in human pancreatic tumors carrying *ATM* mutations. The preferential loss of p16^Ink4A^/p19^Arf^ expression over that of p53 suggests that the need to inactivate the p53/p21 barrier is bypassed in ATM-deficient tumors because p53 is a target of this kinase. In contrast, inactivating p16^Ink4A^/p19^Arf^ is probably necessary in Kras^G12D^-induced tumors to overcome the checkpoints induced by these tumor suppressors and the senescence barrier imposed by the p16^Ink4A^-Rb pathway^21^. Whether genomic instability facilitates the inactivation of p16^Ink4A^/p19^Arf^ in ATM-deficient pancreatic tumor cells or other mechanisms are involved in p16^Ink4A^/p19^Arf^ downregulation warrants further investigation (e.g., ATM loss could enable the DNMT1-mediated methylation of the *p16* ^*Ink4A*^ promoter)^40^.

The results from FISH, gamma-irradiation and qPCR analyses showed that our generated *KCATMΔ*+ pancreatic cancer cells retained a functional wildtype *Atm* allele. These findings are intriguing because in a study reporting germline heterozygous *ATM* mutations in FPC kindreds the analyzed tumors showed *ATM* LOH inactivation^3^. These results argue that transformed human and murine pancreatic cells respond differently to the dosage of ATM, although it is also possible that in our *KCATMΔ*+ tumor cells additional mutations or oncogenic events bypassed the need to inactivate the wildtype *Atm* allele. While these hypotheses remain to be tested, it is noteworthy that similar to our study other investigators reported acceleration of PDGF-induced murine gliomas in the context of *Atm* heterozygosis without LOH^41^.

In addition to providing new information on DNA damage-induced ATM activation in the pancreas, and the effects of ATM deficiency in the context of Kras^G12D^ expression, our study introduces a novel mouse model that should be clinically relevant. First, the pancreatic tumors of our mice have the genetic features of some aggressive forms of human pancreatic cancer, as it was shown that patients carrying tumoral loss of ATM and normal p53 expression tend to have very poor prognosis after tumor resection^42^. Second, the fact that our established ATM-deficient murine pancreatic tumor cells display radiosensitivity is quite relevant because other studies showed that human pancreatic tumor cells become radiosensitized upon treatment with an ATM inhibitor^43^ or *ATM* siRNA^44^. Therefore, our mice are suitable models to test drugs or combined treatments that confer synthetic lethality (e.g., PARP1- or ATR inhibitors^45, 46^) to pancreatic tumors carrying ATM-deficiencies.

In summary, our study determined that the main tumor suppressor role of ATM in the pancreas is to maintain genomic stability. This finding agrees with the canonical genome surveillance role of ATM and has clinical relevance, since human pancreatic tumors that carry *ATM* somatic mutations have ‘unstable genomes’ and are very difficult to overcome. In addition, our discovery that ATM signaling is activated in pancreatic precancerous lesions to prevent the propagation of unrepaired DNA suggests that pathologic conditions that promote DNA damage (e.g., chronic inflammation)^47^ could increase the risk of pancreatic cancer in individuals carrying *ATM* germline mutations.

## Methods

### Mice

Generation of *Atm* ^+/*LoxP*^, *Ptf1a* ^+/*cre*^ and *LSL*-*Kras* ^*G12D*^ mice was described previously^13, 48, 49^. Mice were maintained in a mixed C57BL/6/NMRI genetic background. Animals were monitored at least twice weekly for signs of illness and euthanized when they had early signs of distended abdomen or lethargy. Weight loss was not observed at this stage. At necropsy hemorrhagic ascites and several metastatic nodules in the liver, diaphragm and pleural surfaces (mainly in *KCATMΔ*+ and *KCATMΔΔ* animals) always accompanied the distended abdomen. Mice were treated according to the criteria outlined in the Guide for the Care and Use of Laboratory Animals of the National Institutes of Health. All animal experiments were reviewed and approved by the St Jude Animal Care and Use Committee.

### Human tumor specimens

The patients were enrolled under an IRB approved protocol for bio banking at the University of Michigan, which allows banking the tissue and blood specimens for future research. Patient #1 (46 years old, female, deceased) had metastatic PDA and was treated with gemcitabine/abraxane for 6 months, and subsequently with FOLFOX (Folinic acid, 5-FU, Oxaliplatin), gemcitabine/cisplatin + olaparib and FOLFIRI (Folinic Acid, 5-FU, Irinotecan) in a Phase I clinical trial. A fine needle aspiration biopsy was performed and genomic analysis results identified *ATM* somatic homozygous inactivating mutations (I396fs*10) in the tumor. Patient #2 (66 years old, male) had a borderline resectable PDA and was treated with neoadjuvant FOLFIRINOX (5-FU, leucovorin, irinotecan, oxaliplatin) and gemcitabine plus radiation therapy. A core biopsy of the resected tumor was performed and genomic analysis identified a heterozygous *ATM* germline inactivating mutation (c.4143dupT). All experimental protocols were approved by the University of Michigan Clinical Research Ethics Committee and all procedures were performed in accordance with the approved guidelines and regulations.

### Immunohistochemistry

Immunostaining on paraffin and frozen was performed as previously described^14^. All primary and secondary antibodies used in this study are listed in Supplementary Table 3. Biotinylated anti-rabbit IgG antibodies were detected by using the VECTASTAIN Elite ABC kit (Vector Laboratories). Prolong Gold with DAPI (40,6-diamidino-2-phenylindole; Invitrogen) was used for nuclear staining. Images were obtained with a Zeiss Axioskop 2 microscope, or with a confocal/Multiphoton laser-scanning Zeiss LSM 510 META microscope.

### SA-β-gal/Ki67 staining

Detection of SA-β-gal was performed using published methods^50^. Following this step, sections were post-fixed with 4% PFA (10 min, RT) and processed for immunostaining using anti- Ki67 antibodies. Sections were counterstained with nuclear Fast Red.

### Morphometric analysis

Low and high power confocal images were analyzed using Image J and γH2AX-Ecadherin double positive cells were counted using the “cell counter” plugin. High power confocal images were used to count γH2AX^+^ foci in at least 100 cells from each primary cell line. Low magnification images covering the entire pancreas were captured using the Aperio scanner and Alcian Blue positive lesions were counted using Image J (3 or more sections spanning the whole pancreas were used per genotype).

### Primary cell lines

Primary pancreatic ductal cell cultures were established from *KC*, *KCATMΔ*+ and *KCATMΔΔ* primary and metastatic tumors, according to established protocols^16–18^. Cells were passaged serially 4–5 times to insure homogeneity before analysis. All *in vitro* analyses were performed on low passage number lines (<P8).

### Quantitative PCR and RNA isolation

Total RNA was extracted using TRizol reagent (Thermo Fisher Scientific) and PureLink mini kit (Invitrogen). The purity and integrity of the isolated RNA were determined using a spectrophotometer NanoDrop One/One (Thermo Fisher Scientific). cDNA synthesis was performed using Protoscript first strand cDNA synthesis kit (New England Biolabs) and random hexamers according to the manufacturer’s instructions. iTaq™ Universal SYBR® Green Supermix (Bio-rad) was used for qPCR and the detection was performed in a Mastercycler® RealPlex2 (Eppendorf). Gene expression was normalized against the expression of β-Actin using the Relative Standard Curve Method. Results were expressed as the Mean ± SE (standard error) from three different replicates. The primers used in this study are listed in Supplementary Table 4.

### γ-irradiation of cultured cells

Cells after passage 4 were plated in 24-well plates at a concentration of 50,000 cells per well. The cells were irradiated using a 137 Cs-source (Gammacell 40-exactor, MDS Nordion, Ottawa, Ontario, Canada) at a dose of 4Gy and collected at the designated time points.

### Clonogenic assay

Clonogenic assays on tumor cells exposed to different x-ray doses (RS 2000 X-ray Irradiator, Rad Source) were performed following published methods^51^. Briefly, confluent 100 mm plates of primary tumor cells from *KC*, *KCATMΔ*/+ and *KCATMΔΔ* mice were trypsinized, counted with a hemocytometer and diluted with complete media to obtain 160, 400 or 800 cells per well of each genotype respectively. Colonies were stained with crystal violet after 7 days, and those containing at least 50 cells were counted as surviving colonies. The plating efficiency and the survival fraction for each cell line after each treatment were calculated as described in Franken *et al*.^51^. Survival was calculated in comparison with non-treated samples, using an average of three determinations of the same dose rate of cells (±SE).

### Western blot

Cells (~800,000) from primary tumor and liver metastasis were seeded in 60 mm plate dishes. After 22 hours the culture medium was changed and 2 hours later the cells were irradiated (4Gy) using an RS 2000 X-ray Irradiator (Rad Source). Cell extracts were collected at 0, 1, 4 and 24 hours post-irradiation. Cells were harvested in cold PBS, and then lysed in 250 µL of RIPA Buffer (Sigma Aldrich). Protein quantification was performed using the Bradford reagent (Bio Rad) and samples were prepared by adding NuPAGE LDS Sample Buffer (Thermo Fisher Scientific) and β -mercaptoethanol. 30 µg of whole cell extracts were loaded into 10% NuPAGE Bis-Tris gel (Thermo Fisher Scientific) and electrophoresed at 110 volts in NuPAGE MES SDS Running Buffer (Thermo Fisher Scientific). Proteins were transferred to PDVF membrane using 10% Methanol NuPAGE Transfer Buffer (Thermo Fisher Scientific) for 16 hours at 30 volts and 4 °C. Primary antibodies were incubated 2 hours at room temperature and secondary donkey Horseradish Peroxidase-conjugated antibodies (Jackson) were incubated 1 hour at room temperature. Membranes were developed using SuperSignal™ West Femto Maximum Sensitivity Substrate (Thermo Fisher Scientific). The primary antibodies used here are listed in Supplementary Table 3. Three independent hybridizations were performed for each primary antibody. The corresponding bands were scanned and quantified by densitometry using the CLIQS software from TotalLab (www.http://totallab.com/). The median ratio of p-p53/total p53, p-Kap1/total Kap1 or p21/β-actin from the three experiments ± SE (standard error) was calculated.

### Histochemistry

Alcian Blue staining for visualization of PanINs’ mucinous content and Picrosirius Red staining for collagen fibers were performed according to manufacturer’s instructions (Abcam). Immunohistochemically and histochemically stained slides were further scanned with an Aperio® slide scanner (Leica).

### Immunofluorescence of cultured cells

Cells were cultured, fixed and stained as previously described^14^.

### Fluorescence *in situ* hybridization (FISH)

Cells were grown to 60–70% confluence, treated with colcemid (0.02 µg/mL) for a 4 h incubation at 37 °C, and harvested using routine cytogenetic methods. The cells were then trypsinized for 5 minutes at 37 °C and centrifuged at 900 RPM for 5 minutes. The cell pellet was resuspended in 0.075 M KCL for 8 minutes at RT and then centrifuged at 600 RPM for 5 minutes, resuspended in 3:1 Carnoy’s fixative (3 parts methanol: 1 part acetic acid), and incubated for 15 minutes at RT. The cells were centrifuged at 600 RPM for 5 minutes, resuspended in Carnoy’s fixative, and incubated for 10 minutes at RT. The cells were spread onto a glass slide using a Pasteur pipette. The slides were air dried at RT. Purified DNA from *Atm* (RP23-456L9/9A5.3) was labeled with a red-dUTP (AF594, Molecular Probes) and purified DNA from a chromosome 9 control (RP23-284E19/9A5.2) was labeled with a green-dUTP (AF488, Molecular Probes) by nick translation. The probes were combined and hybridized to interphase and metaphase cells derived from the two samples using routine cytogenetic methods. Cells were stained with DAPI and total of one hundred interphase nuclei were scored for the number of red and green signals per cell.

### Spectral Karyotype (SKY) Analysis

Slides containing cells in metaphase were prepared as described before. Spectral karyotyping of chromosomes was performed using a mouse SkyPaint probe (Applied Spectral Imaging, Carlsbad, CA) per the manufacturer’s recommendation. Probes were detected using Applied Spectral Imaging’s concentrated antibodies detection kit (CAD), as described by the manufacturer. Cells were stained with 4, 6-diamidino-2-phenylindole (DAPI) and images were acquired with a Nikon Eclipse E600 fluorescence microscope equipped with an interferometer (Spectra Cube: Applied Spectral Imaging) and a custom designed filter cube (Chroma Technology Corporation, Rockingham, VT). HiSKY software version 7.2 (Applied Spectral Imaging) was used for SKY analysis.

### Mutation analyses of *Trp53* and *Cdkn2a*

For *Trp53* exon sequence analysis, genomic DNA was isolated from 3 *KC* and 3 *KCATMΔΔ* primary cell lines (pancreatic tumor and liver metastases, <P8) using the Wizard DNA purification kit (Promega). For *Trp53*, *p16* ^*Ink4a*^ and *p19* ^*Arf*^ cDNA sequencing, total RNA was extracted and cDNA was synthesized as described^13^. Accuprime high fidelity polymerase (Life Sciences) was used for PCR. PCR products were purified using the QIAquick gel extraction kit (Qiagen science, Maryland, USA), and submitted for sequencing using the same forward and reverse primers used for amplification. Supplementary Table 5 lists the primers used for this analysis.

### MTT assay

Human cell lines and murine PDAC and liver metastasis primary cell lines maintained in the logarithmic phase of growth were trypsinized, counted, and plated at 100 cells/well in 96 well plates in a total volume of 100 ml of complete medium. Twenty-four hours later, cells were irradiated and viability was assessed starting on the same day and for 6–7 more days using the Cell Titer Aqueous Viability assay (Promega) following the manufacturer’s instructions. Cell titer aqueous is based on an improved version of the standard MTT assay where the MTS tetrazolium compound is bioreduced by cells into a colored formazan product that is soluble in tissue culture medium in contrast to medium-insoluble MTT product. Therefore, the colorimetric assessment of proliferation can be performed in one step.

### Statistical Analyses

In all graphs, data are presented as mean ± SEM. Significance was accepted at a *P* value < 0.05. PRISM software was used to perform statistical analysis for all datasets. For survival analysis the Log-rank (Mantel-Cox) test was performed. For comparison of Alcian Blue or γH2AX staining results in the 3 genotypes, ordinary ANOVA one-way was performed. For Clonogenic and MTS data from different time points, ANOVA 2-way analysis was performed with Bonferroni’s test for multiple comparisons.

## Electronic supplementary material

Supplementary Information
