# Supplementary material for: ATM-deficiency increases genomic instability and metastatic potential in a mouse model of pancreatic cancer
Source: Sci Rep. 2017 Sep 11;7:11144. doi: 10.1038/s41598-017-11661-8 (PMC5593966; doi:10.1038/s41598-017-11661-8)
Supplement: Supplementary file 1 — Supplementary Information [file 41598_2017_11661_MOESM1_ESM.pdf]

ATM-deficiency increases genomic instability and metastatic potential in a mouse model of pancreatic cancer

Yiannis Drosos, David Escobar, Ming-Yi Chiang, Kathryn Roys, Virginia Valentine, Marc B. Valentine, Jerold E. Rehg, Vaibhav Sahai, Lesa A. Begley, Jianming Ye, Leena Paul, Peter J. McKinnon, Beatriz Sosa-Pineda.

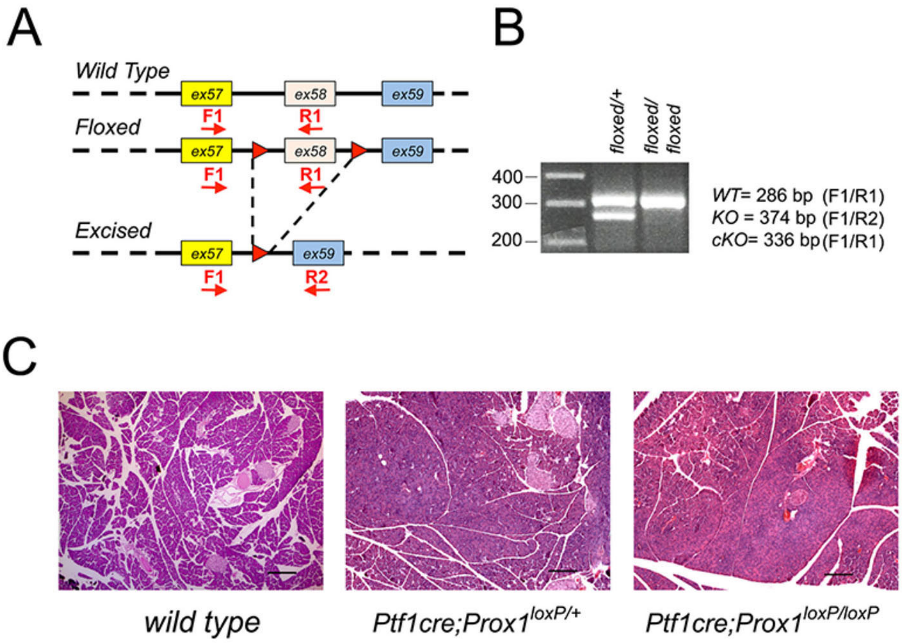

Supplementary Figure 1. Conditional deletion of *Atm* in the pancreas. **A**: Schematic representation of the *Atm* locus in *wildtype*, *Atm*^{LoxP} (Floxed allele), and *Ptf1a-Cre*;*Atm*^{LoxP} (Excised allele). Exons 57, 58 and 59 in the *Atm* coding (kinase domain) region are shown with colored boxes. Under the activity of Cre recombinase exon 58 is excised, resulting in a kinase-deficient ATM protein. **B**: Primers F1 and R1 amplify a 286 bp product from the *wildtype* allele and a 336 bp product from the *LoxP*-containing allele ("*Floxed*"). Primers F1 and R2 amplify a 374 bp sequence from the *excised* allele. **C**: H&E staining shows that pancreas histology is comparable amongst *wildtype*, *Ptf1-Cre;Atm*$^{LoxP/+}$ (*Atm* pancreas heterozygous) and *Ptf1-Cre;Atm*$^{LoxP/LoxP}$ (*Atm* pancreas-homozygous) adult mice. Scale bars: 100μm.

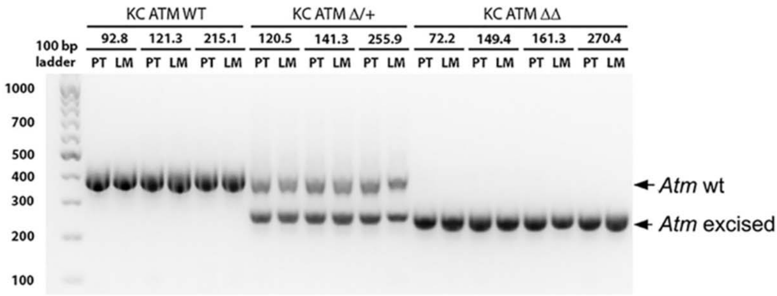

		Norm. expr.(10^{-5})	SD (10^{-5})
KC	92.8	1.4	9.6
	121.3	0.2	2.8
	215.1	0.7	4.8
KCAΔ+	120.5	43.1	4.4
	141.3	49.1	4.2
	255.9	119.0	42.6
KCAΔΔ	72.2	61.9	0.5
	149.4	32.6	0.1
	161.3	46.7	0.4

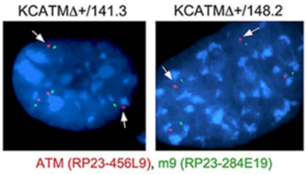

Supplementary Figure 2. *KCATMΔ*+ tumor cell lines do not show Loss of Heterozygosity (LOH). **A**: RT-PCR results using cDNA isolated from *KC*, *KCATMΔ*+ and *KCATMΔΔ* primary tumor cell lines and primers spanning exon 58 (Forward: TGTGGGTTCTGATGGCAAGGAAAG. Reverse: CAAACCAAACAGCTGGGTCCAAGA). PCR products of the expected sizes from both the *Atm* wildtype allele and the *Atm* (exon 58) floxed allele are amplified from all the *KCATMΔ*+ samples. **B**: qPCR results showing the abundance of a fragment spanning exon 58 relative to that of beta-actin in PDAC cell lines of the 3 genotypes. **C**: FISH analysis shows no evidence of large deletions in the *Atm* locus in 2 independent primary *KCATMΔ*+ tumor cell lines (141.3 and 148.2). The red probe hybridizes to a region in chromosome 9 where *Atm* is located; the green probe hybridizes to a region in chromosome 9 outside the *Atm* locus.

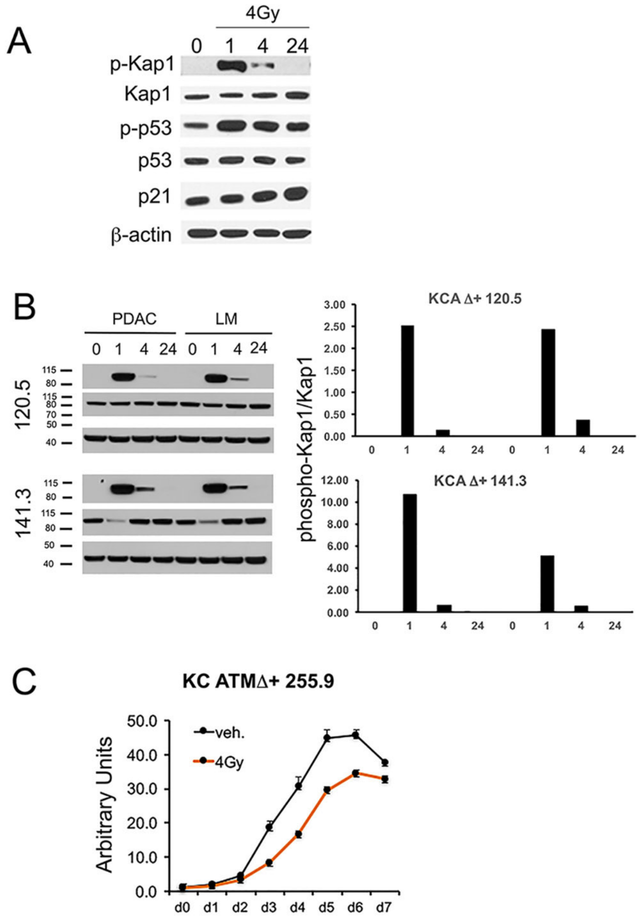

Supplementary Figure 3. *KCATMΔ+* tumor cells show activation of signals downstream of ATM upon irradiation. **A**: Western blots show induction of phospho-Kap1, phospho-p53 and p21 in MEFs upon 4Gy IR exposure. **B**: (*left*) Western blots showing phospho-Kap1 induction 1-4hr post-irradiation in *KCATMΔ+* 120.5 and 141.3 tumor cells. (*right*) Quantification of Western blot results by densitometry. **C**: *KCATMΔ+* 255.9 (PDAC) cells do not display significant radiosensitization in an MTT assay. (Error bars represent ± SEM values of triplicate experiments, 2-Way ANOVA.)

Supplementary Table 1. Survival group tumor analysis

	ID	Genotype	Age (days/months)	Pancreatic tumor	Metastasis			Ascites	Other
					Liver	Diaphragm	Lung		
1	120.1	**KCATMΔΔ**	140/5 (E)	Y	Y	Y	Y	Y	
2	149.4		154/5 (E)	Y	Y	Y	N	Y	Peritoneum
3	80.6		83/6 (E)	Y	Y	NA	NA	NA	
4	120.4		200/7 (E)	Y	Y	N	N	Y	
5	270.4		222/7 (FD)	Y	Y	N	N	NA	
6	162.3		199/7 (FD)	Y	Y	N	N	NA	
7	161.3		239/8 (E)	Y	Y	N	N	Y	
8	270.4		222/8 (E)	Y	Y	N	N	N	
9	111.4		239/8 (FD)	Y	Y	N	N	NA	
10	72.2		263/9 (E)	Y	N	N	N	N	
11	280.4		275/9 (FD)	Y	Y	N	N	NA	
12	72.4		252/10 (E)	Y	N		N	Y	
13	164.4		281/10 (FD)	N	N	N	N	NA	
14	155.6		276/10 (FD)	Y	Y	N	N	NA	
15	72.6		293/10 (FD)	Y	Y	N	N	NA	
16	162.4		318/11 (E)	Y	Y	N	N	Y	Obstructed bile duct, jaundice
17	73.7		348/12 (E)	Y	Y	N	N	Y	
18	98.4		361/12 (E)	Y	Y	N	N	Y	Liver cirrhosis
1	141.8	**KCATMΔ+**	90/3.5 (E)	N	N	N	N	N	Extensive inflammation
2	111.8		150/5 (E)	N	N	N	N	N	Hind limb paralysis, ataxia, spinal tumor
3	115.1		180/6 (E)	N	N	N	N	N	Hypoglycemia
4	141.3		229/8 (E)	Y	Y	N	N	Y	
5	148.2		223/8 (E)	Y	Y	N	N	Y	
6	111.1		240/8 (FD)	Y	Y	N	N	NA	
7	255.9		254/9 (E)	Y	Y	N	N?	Y	Cysts
8	167.9		274/9 (FD)	Y	Y	N	N	NA	
9	148.4		275/9 (FD)	Y	Y	N	N	NA	
10	120.7		273/9 (FD)	N	N	N	N	NA	
11	172.6		291/10 (FD)	N	N	N	N	NA	
12	133.1		331/11 (FD)	Y	Y	N	N	NA	
13	161.8		324/11 (FD)	Y	Y	N	N	NA	
14	167.8		321/11 (FD)	N	N	N	N	NA	
15	167.6		364/12 (FD)	N	N	N	N	NA	Cysts
16	120.3		424/14 (FD)	Y	Y	N	N	NA	
17	121.4		404/14 (FD)	Y	Y	N	N	NA	
18	120.5		469/16 (E)	Y	Y	Y	N	Y	
19	165.1		474/16 (E)	Y	Y	Y	Y	Y	
20	121.1		517/17 (E)	N	N	N	N		Cysts
21	193.7		516/17 (E)	Y	Y	N	Y	Y	
1	92.8	**KC**	201/7 (E)	Y	Y	N	N	Y	
2	215.1		207/7 (E)	Y	Y	N	N	Y	
3	88.8		274/10 (E)	N	N	N	N	N	
4	309.1		294/10 (FD)	Y	N	N	N	NA	
5	14226<br>40.2		304/10 (FD)	N	N	N	N	NA	
6	14226<br>36.1		330/11 (FD)	Y	N	N	N	NA	
7	14226<br>36.3		375/12 (FD)	N	N	N	N	NA	
8	121.3		375/13 (E)	Y	Y	N	N	Y	Liver cirrhosis
9	72.5		384/13 (E)	N	N	N	N	N	Cysts
10	285.8		409/14 (E)	N	N	N	N	N	

11	147.1	466/15 (FD)	N	N	N	N	NA
12	92.4	451/15 (FD)	N	N	N	N	NA
13	80.1	498/16 (FD)	Y	Y	N	N	NA
14	97.5	492/16 (FD)	N	N	N	N	NA
15	14226<br>36.2	527/17 (FD)	N	N	N	N	NA
16	13430<br>97.8	528/18 (FD)	N	N	N	N	NA
17	27.6	508/19 (FD)	Y	N	N	N	NA
18	97.9	610/21 (E)	Y	Y	Y	N	Y
19	132.8	718/24 (FD)	N	N	N	N	NA

E: Euthanized
FD: Found dead
NA: Not Applicable

Supplementary Table 2. QPCR results showing CT values normalized to β-actin.

			PROGENITOR										SQUAMOUS				IMMUNOGENIC				ADEX			
			Lgals4		*Hnf1B*		*Gata6*		*Pdx1*		*HNF4a*		*Egfr*		*Prrx1*		*Igll1*		*Igj*		*Rbpjl*		*Cpa2*	
			CT ave	CT STD	CT ave	CT STD	CT ave	CT STD	CT ave	CT STD	CT ave	CT STD	CT ave	CT STD	CT ave	CT STD	CT ave	CT STD	CT ave	CT STD	CT ave	CT STD	CT ave	CT STD
**KC**	92.8	PT	4.11. E-05	1.07. E-05	6.40. E-04	1.67. E-04	1.41. E-02	3.91. E-03	2.83. E-03	7.38. E-04	1.93. E-07	6.73. E-08	8.91. E-04	2.40. E-04	7.01. E-03	1.49. E-03	2.20. E-07	5.82. E-08	6.49. E-07	1.85. E-07	0.00. E+00	0.00. E+00	0.00. E+00	0.00. E+00
		LM	3.38. E-05	8.20. E-06	2.01. E-03	4.86. E-04	7.63. E-02	1.84. E-02	6.34. E-03	1.53. E-03	8.99. E-07	3.99. E-07	9.05. E-04	2.18. E-04	8.90. E-03	5.13. E-04	2.09. E-07	5.74. E-08	5.99. E-07	2.20. E-07	8.67. E-08	8.64. E-08	0.00. E+00	0.00. E+00
	121.3	PT	1.93. E-02	2.81. E-03	4.40. E-03	6.11. E-04	6.72. E-01	9.72. E-02	1.04. E-02	1.47. E-03	1.38. E-05	2.17. E-06	4.48. E-04	6.23. E-05	3.03. E-06	1.46. E-07	1.80. E-07	3.10. E-08	2.09. E-07	3.48. E-08	1.91. E-08	1.36. E-08	0.00. E+00	0.00. E+00
		LM	3.28. E-02	1.05. E-02	1.45. E-02	1.66. E-03	1.17. E+00	1.34. E-01	2.51. E-02	2.87. E-03	4.51. E-05	5.25. E-06	8.70. E-04	9.96. E-05	1.36. E-05	1.32. E-06	1.51. E-06	2.00. E-07	9.52. E-07	1.29. E-07	0.00. E+00	0.00. E+00	0.00. E+00	0.00. E+00
	215.1	PT	7.45. E-04	1.75. E-04	1.61. E-03	2.72. E-04	4.77. E-02	8.04. E-03	3.67. E-03	6.20. E-04	3.45. E-06	7.30. E-07	1.49. E-04	2.50. E-05	6.81. E-05	3.93. E-06	1.28. E-07	3.23. E-08	4.63. E-08	9.33. E-09	0.00. E+00	0.00. E+00	0.00. E+00	0.00. E+00
		LM	4.36. E-03	8.69. E-04	1.22. E-02	1.08. E-03	7.84. E-01	9.41. E-02	2.41. E-02	2.18. E-03	5.75. E-05	5.14. E-06	1.01. E-03	9.90. E-05	2.85. E-04	1.07. E-05	3.94. E-06	3.94. E-07	4.90. E-07	1.25. E-07	0.00. E+00	0.00. E+00	0.00. E+00	0.00. E+00
**KC ATM ∆/+**	120.5	PT	1.53. E-02	9.64. E-04	1.14. E-02	1.63. E-04	1.15. E+00	2.89. E-02	1.65. E-02	1.50. E-04	7.34. E-05	1.01. E-06	2.67. E-03	1.63. E-04	8.88. E-06	1.16. E-06	1.31. E-06	1.06. E-07	3.88. E-07	1.13. E-07	0.00. E+00	0.00. E+00	0.00. E+00	0.00. E+00
		LM	2.71. E-02	1.11. E-03	5.48. E-03	2.85. E-04	6.34. E-01	2.60. E-02	1.36. E-02	5.56. E-04	4.55. E-05	1.82. E-06	8.38. E-04	3.33. E-05	2.11. E-05	1.92. E-06	4.58. E-07	8.33. E-08	2.47. E-07	5.76. E-08	8.14. E-08	4.48. E-08	1.63. E-07	3.04. E-08
	141.3	PT	3.40. E-03	4.61. E-04	1.06. E-02	1.06. E-04	7.96. E-01	2.54. E-02	2.07. E-02	2.79. E-04	6.00. E-05	5.82. E-06	1.83. E-03	3.87. E-05	5.41. E-05	2.37. E-06	8.12. E-08	3.37. E-08	6.13. E-07	3.31. E-08	3.18. E-07	9.59. E-08	0.00. E+00	0.00. E+00
		LM	6.70. E-04	2.17. E-04	6.64. E-03	1.41. E-03	2.37. E-01	5.06. E-02	1.13. E-02	2.41. E-03	3.54. E-05	7.60. E-06	9.58. E-04	2.10. E-04	4.96. E-05	4.55. E-06	2.71. E-07	5.89. E-08	5.33. E-07	1.53. E-07	1.34. E-07	2.86. E-08	4.40. E-08	1.18. E-08
	255.9	PT	3.99. E-04	3.71. E-05	4.82. E-02	4.91. E-03	6.87. E-01	5.96. E-02	3.46. E-02	2.93. E-03	5.13. E-05	6.07. E-06	1.34. E-03	1.30. E-04	5.10. E-05	1.03. E-06	1.03. E-06	1.58. E-07	1.62. E-06	3.89. E-07	1.82. E-07	3.45. E-08	0.00. E+00	0.00. E+00
		LM	7.44. E-03	3.68. E-04	5.26. E-02	5.81. E-04	3.91. E-01	3.89. E-03	1.85. E-02	2.03. E-04	1.32. E-05	5.02. E-07	4.40. E-04	2.30. E-05	5.95. E-06	3.85. E-07	7.62. E-07	1.73. E-08	3.70. E-07	1.40. E-07	2.28. E-07	7.92. E-08	0.00. E+00	0.00. E+00
**KC ATM ∆∆**	72.2	PT	2.83. E-02	1.38. E-03	1.01. E-02	4.56. E-04	2.20. E+00	2.12. E-01	1.15. E-02	5.19. E-04	9.03. E-05	1.31. E-05	1.87. E-03	8.57. E-05	1.71. E-04	1.56. E-05	2.80. E-06	1.60. E-07	1.19. E-06	3.05. E-07	2.49. E-07	1.27. E-08	0.00. E+00	0.00. E+00
		LM	9.71. E-03	1.08. E-03	1.04. E-02	1.12. E-03	7.67. E-01	8.22. E-02	1.62. E-02	1.69. E-03	4.05. E-05	4.22. E-06	9.35. E-04	9.75. E-05	1.09. E-04	1.41. E-05	1.74. E-06	1.85. E-07	6.70. E-07	8.89. E-08	1.15. E-07	4.61. E-08	1.12. E-07	4.40. E-08
	149.4	PT	4.48. E-03	2.34. E-04	2.96. E-03	1.25. E-04	2.85. E-01	1.43. E-02	7.73. E-03	3.07. E-04	1.35. E-05	5.67. E-07	2.38. E-04	1.55. E-05	8.14. E-06	3.29. E-07	6.21. E-07	3.53. E-08	2.42. E-07	3.97. E-08	0.00. E+00	0.00. E+00	0.00. E+00	0.00. E+00
		LM	8.25. E-03	2.08. E-03	4.51. E-03	1.07. E-03	1.72. E-01	4.07. E-02	7.88. E-03	1.88. E-03	9.68. E-06	2.32. E-06	3.09. E-04	7.34. E-05	6.81. E-06	9.52. E-07	3.47. E-07	9.23. E-08	2.06. E-07	9.40. E-08	0.00. E+00	0.00. E+00	0.00. E+00	0.00. E+00
	161.3	PT	6.66. E-02	1.30. E-02	6.12. E-03	1.15. E-03	1.40. E+00	2.63. E-01	2.04. E-02	3.95. E-03	3.36. E-05	8.41. E-06	1.10. E-03	2.06. E-04	4.53. E-05	2.07. E-06	5.87. E-07	1.96. E-07	5.75. E-07	1.53. E-07	3.94. E-08	4.35. E-08	1.33. E-07	2.65. E-08
		LM	1.26. E-01	3.26. E-02	2.68. E-02	5.55. E-03	8.69. E-01	1.82. E-01	2.27. E-02	4.75. E-03	7.29. E-05	1.76. E-05	7.84. E-04	1.63. E-04	3.03. E-04	1.50. E-06	1.94. E-06	6.48. E-07	8.09. E-07	2.23. E-07	0.00. E+00	0.00. E+00	0.00. E+00	0.00. E+00
	270.4	PT	3.88. E-03	2.43. E-04	5.89. E-03	2.73. E-04	1.95. E-01	1.33. E-02	6.61. E-03	2.65. E-04	1.73. E-05	2.03. E-06	2.78. E-04	1.11. E-05	2.25. E-05	2.65. E-06	3.64. E-07	5.19. E-08	1.97. E-07	3.58. E-08	2.19. E-08	3.71. E-09	6.52. E-08	3.16. E-08
		LM	3.92. E-03	6.85. E-04	7.01. E-03	1.22. E-03	1.82. E-01	3.17. E-02	7.13. E-03	1.23. E-03	9.63. E-06	1.68. E-06	2.62. E-04	4.57. E-05	6.77. E-05	1.92. E-06	1.92. E-07	3.32. E-08	1.83. E-07	4.48. E-08	0.00. E+00	0.00. E+00	0.00. E+00	0.00. E+00
**PCRS.**																					8.90. E-03	3.58. E-04	6.74. E-01	7.38. E-03

Supplementary Table 3. Primary and secondary antibodies used in the study

Primary antibodies

**Antigen**	**Species**	**Source**	**Catalogue number**	**Paraffin (P)<br>Frozen (F)<br>Cultured cells (C)<br>Western blot (WB)**	**Dilution**
β-actin	Mouse	Sigma	A2228	WB	1:100,000
TROMA-III/CK19*	Rat	DSHB, U. of Iowa		P	1:100
F4/80	Rat	ABD Serotech	MCA497GA	1:1,000	P
γH2AX	Rabbit	Cell Signaling	9718	P<br>F, C	1:500<br>1:100
KAP1	Rabbit	Bethyl	A300-274A	P, F, C<br>WB	1:1,000<br>1:2,000
Phospho-KAP1	Rabbit	Bethyl	A300-767A	P, F, C<br>WB	1:100<br>1:250
Ki67	Rabbit	Thermo	RM-9106	P, F, C	1:500
p16Ink4a	Rabbit	Santa Cruz	SC-1207	P, C	1:100
p19/Arf	Rat	LifeSpan	LS-C2744	P, C	1:500
p21	Mouse	Santa Cruz	SC-6246	P<br>WB	1:100<br>1:500
p53	Rabbit	Proteintech	10442-1-AP	WB	1:1,000
Phospho-p53	Rabbit	Cell Signaling	9284	WB	1:250
Hnf1β	Rabbit	ProtechGroup	12533-1-AP	P	1:5,000

Secondary antibodies

**Antigen**	**Species**	**Source**	**Catalogue number**	**Dilution**	**Conjugate**
Rabbit IgG	Donkey	Jackson Lab	711-065-152	1:500	Biotinylated
Rat IgG	Goat	Jackson Lab	112-065-003	1:500	Biotinylated
Sheep IgG	Donkey	Jackson Lab	713-065-003	1:500	Biotinylated
Rabbit IgG	Donkey	Jackson Lab	711-165-152	1:500	Cyanine Cy-3
Rabbit IgG	Donkey	Molecular Probes	A21206	1:500	Alexa-488
Rat IgG	Donkey	Molecular Probes	A21208	1:500	Alexa-488
Sheep IgG	Donkey	Jackson Lab	713-165-003	1:500	Cyanine Cy-3

*The TROMA-III antibody developed by R. Kemler was obtained from the Developmental Studies Hybridoma Bank, created by the NICHD of the NIH and maintained at The University of Iowa, Department of Biology, Iowa City, IA 52242.

Supplementary Table 4. qRT-PCR primers used for tumor classification

Gene		5' – 3'
Igj	Forward Primer	TGACGACGAAGCGACCATTC
	Reverse Primer	TTCAAAGGGACAACAATTCGGA
Igll1	Forward Primer	ACTCTGTTCCTGCCTTCCTTA
	Reverse Primer	GAGGGTTGGGTTGTCTCTACA
Cpa2	Forward Primer	AAGAGCACCTTGAGCTTGATTT
	Reverse Primer	GAACTCGGACATGGACTGTCT
Rbpjl	Forward Primer	ACCGTCTGCGGTTACATGG
	Reverse Primer	GGTCTTGGCACAACCAAATTC
Egfr	Forward Primer	GCCATCTGGGCCAAAGATACC
	Reverse Primer	GTCTTCGCATGAATAGGCCAAT
Lgals4	Forward Primer	GGTCGTGGTGAACGGAAATTC
	Reverse Primer	GTGGAGGGTTGTACCCAGGA
Prrx1	Forward Primer	AGCAGACGAAAGTGTGGGC
	Reverse Primer	TCAGAGTTCAACTGGTCATTGTC
Trp53	Forward Primer	CTTACCAGGGCAACTATGGCTT
	Reverse Primer	GCCAGCTGGCAGAATAGCTTATTG
p19 Arf	Forward Primer	GCTCTGGCTTTCGTGAACATGTTG
	Reverse Primer	TTCGAATCTGCACCGTAGTTGAGC
p16 Ink4a	Forward Primer	AACTCTTTCGGTCGTACCCCGATT
	Reverse Primer	ATCTGCACCGTAGTTGAGCAGAAG
Gata6	Forward Primer	GCTCTGTCCCTATGACTCCTACTT
	Reverse Primer	TTCTCCCACTGCAGACATCACT
Hnf4a	Forward Primer	CCTTATGCCCTCACAGGGTC
	Reverse Primer	GACATCCTCCTCCTGGTCCT
Hnf1b	Forward Primer	AGGGAGGTGGTCGATGTCA
	Reverse Primer	TCTGGACTGTCTGGTTGAACT
Pdx1	Forward Primer	AGCTCCCTTTCCCGTGGATGAAAT
	Reverse Primer	AGGCAGTACGGGTCCTCTTGTTTT
B-actin	Forward Primer	AGATCAAGATCATTGCTCCTCCT
	Reverse Primer	ACGCAGCTCAGTAACAGTCC

Supplementary Table 5. RT-PCR primers used for sequencing

Description	Forward primer (5'-3')	Reverse primer (5'-3')
Trp53 Exons 2-3 (set1)	ACCCTTTCCTATAAGCCATAG	GTCCATGCAGTGAGGTGATGG
Trp53 Exons 2-3 (set2)	ATGACTGCCATGGAGGAGTCA	CAGGATATCTTCTGGAGGAAG
Trp53 Ex. 4	TGGTAAGGCCCAGAGCAGAA	GAGATGCAGAGAATATGAGA
Trp53 Ex. 5	AATGGTGCTTGGACAATGTGTTTCA	ACCCGGATAAGATGCTGGGGA
Trp53 Ex. 6	CGATGGTGATGGTAAGCCCTCAA	TAGCTAGCACTCAGGAGGGT
Trp53 Ex. 7	AGAGGCTATAGCCAGCCATT	GGAACCAAAGAGCGTTGGGCAT
Trp53 Ex. 8	TCTTATCTGTGGCTTCTCGG	TTGTGCAGGTGGGCAGCGCTGTGG
Trp53 Ex. 9	GAAGTCCTTTGCCCTGAACT	TTTTATACATGCGAGAGACA
Trp53 Ex. 10	ACCTGTAAGTGGAGCCAGCT	AGCAGGGGTGGGGTTTTTATC
Trp53 Ex.11	CCAGCCTAGAGCCTTCCAAGCCTTGA	TTGTGTCTCAGCCCTGAAGTCATAAGA
Trp53 cDNA	GCAGGGTGTCACGCTTCTCCG	GGCTGGTGATGGGGACGGGA
Cdkn2a Ex. 1a (specific for p16)	AGCAGTGTTTTCAGGGGTGT	CAGATGGGACACTCCTTGCC
Cdkn2a Ex. 1b (specific for p19)	AAGTTAACCGGAGCGAAAGC	CATCTCCCGGGAACTAAGCC
Cdkn2a Ex. 2	AGGCAAGTGCTGTGTGTCTA	TCAGGCTCATTTGGGTTGCT

Cdkn2a Ex. 3	GAGCCTAGCAAAGGGGGTTG	ATGGGATGGGGAACCAGAGT
p16^{Ink4a} cDNA	AACTCGAGGAGAGCCATCTG	GCCACATGCTAGACACGCTA
p19^{Arf} cDNA	CTTCTCACCTCGCTTGTCAC	AGCTCTGCTCTTGGGATTGG

Experiment 1

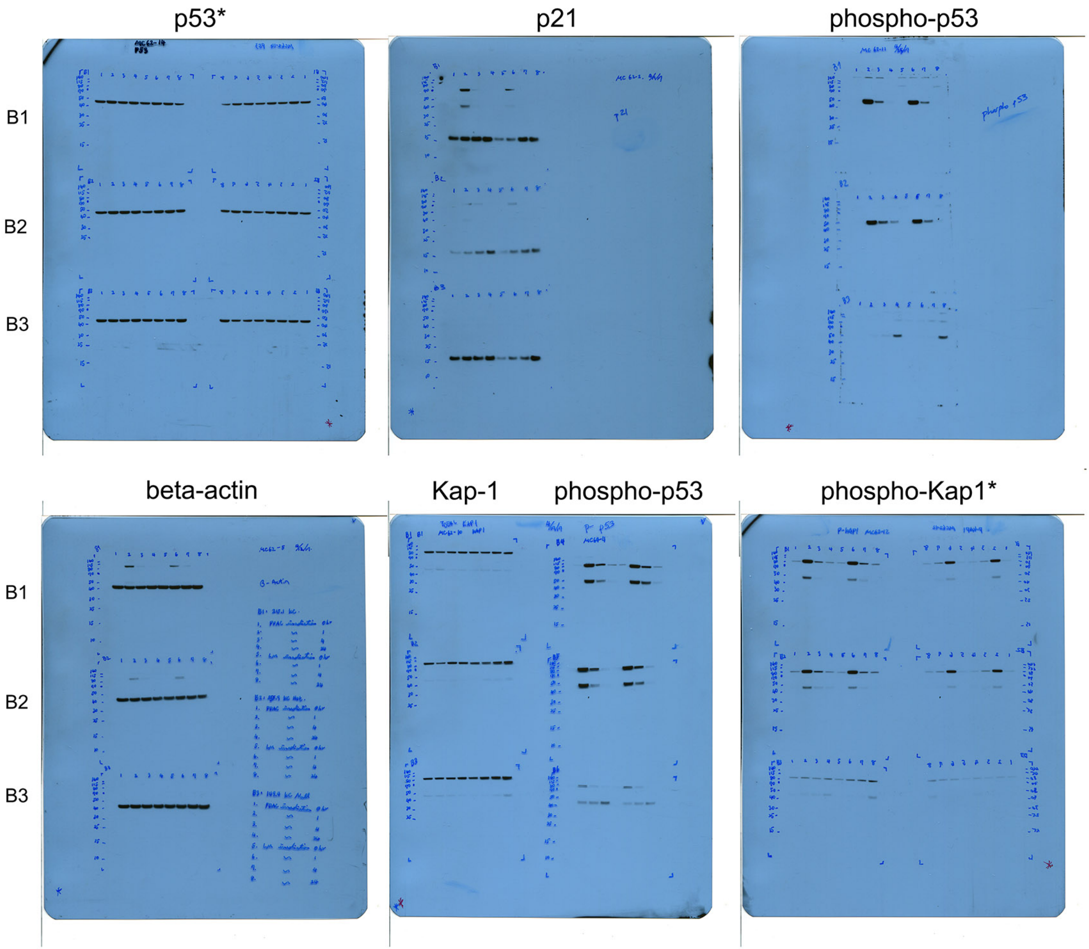

* Two differente exposures are shown to the left and right.

Experiment 2

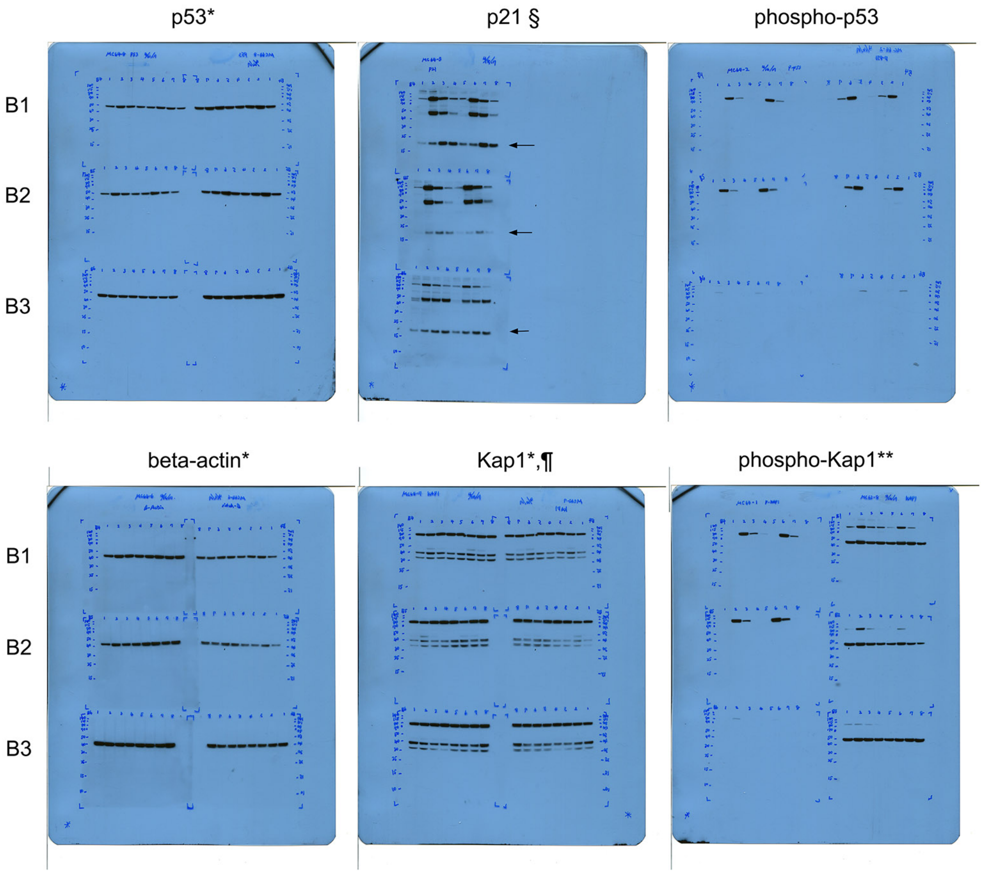

* Two exposures (left and right).

** Two exposures; membrane to the right was stripped twice and hybridized for p53 and Kap1.

§ Membrane was hybridized for p21 (arrow) and then stripped twice and hybridized for p-Kap1 and p-p53.

¶ The 30-40 kd doublet is unspecific.

Experiment 3

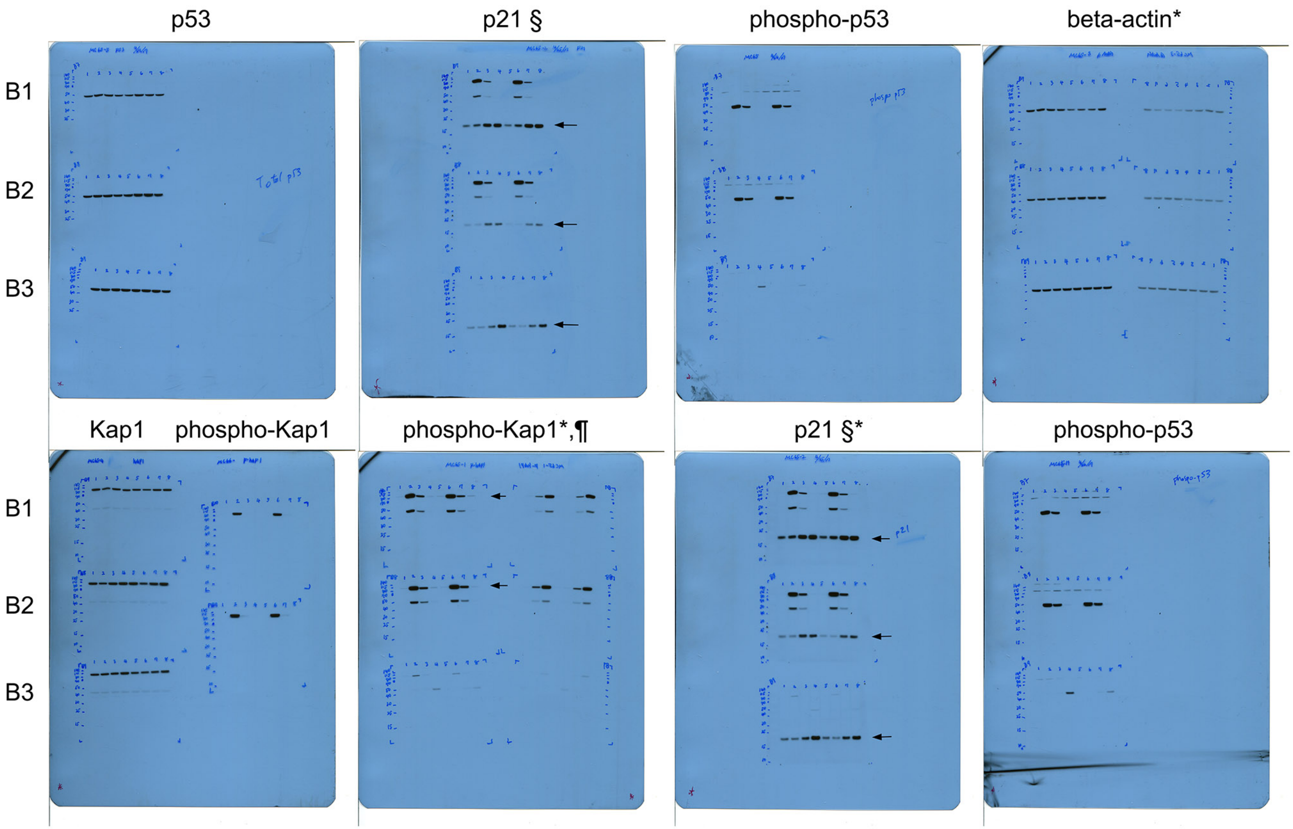

* Different exposures (left and right)

§ Membrane was hybridized for p-Kap1 and p-p53 (upper bands), stripped and hybridized for p21 (arrows).

¶ Membrane was hybridized for p-Kap1 (arrows), stripped and hybridized for p-p53

Western Blot samples

B1: KC 215.1
B2: 255.9 KCAD+
B3: 149.4 KCADD

PDAC: primary tumor cells
LM: liver metastasis cells

Cell were irradiated (4 Gy X-ray) and harvested at different time points. This experiment was repeated 3 times.

1. PDAC, 0hr
2. PDAC, 1hr
3. PDAC, 4hr
4. PDAC, 12hr
5. LM, 0hr
6. LM, 1hr
7. LM, 4hr,
8. LM, 24hr
